# Modeling SILAC Data to Assess Protein Turnover in a Cellular Model of Diabetic Nephropathy

**DOI:** 10.3390/ijms24032811

**Published:** 2023-02-01

**Authors:** Barbara Di Camillo, Lucia Puricelli, Elisabetta Iori, Gianna Maria Toffolo, Paolo Tessari, Giorgio Arrigoni

**Affiliations:** 1 Department of Information Engineering, University of Padova, 35131 Padova, Italy; 2 Department of Medicine, University of Padova, 35128 Padova, Italy; 3 Proteomics Center, University of Padova and Azienda Ospedaliera di Padova, 35128 Padova, Italy; 4 Department of Biomedical Sciences, University of Padova, 35131 Padova, Italy

**Keywords:** proteomics, protein turnover rate, protein half-life, SILAC, diabetes, nephropathy, fibroblasts

## Abstract

Protein turnover rate is finely regulated through intracellular mechanisms and signals that are still incompletely understood but that are essential for the correct function of cellular processes. Indeed, a dysfunctional proteostasis often impacts the cell’s ability to remove unfolded, misfolded, degraded, non-functional, or damaged proteins. Thus, altered cellular mechanisms controlling protein turnover impinge on the pathophysiology of many diseases, making the study of protein synthesis and degradation rates an important step for a more comprehensive understanding of these pathologies. In this manuscript, we describe the application of a dynamic-SILAC approach to study the turnover rate and the abundance of proteins in a cellular model of diabetic nephropathy. We estimated protein half-lives and relative abundance for thousands of proteins, several of which are characterized by either an altered turnover rate or altered abundance between diabetic nephropathic subjects and diabetic controls. Many of these proteins were previously shown to be related to diabetic complications and represent therefore, possible biomarkers or therapeutic targets. Beside the aspects strictly related to the pathological condition, our data also represent a consistent compendium of protein half-lives in human fibroblasts and a rich source of important information related to basic cell biology.

## 1. Introduction

Modern technological advances have provided investigators with sophisticated methodologies that allow an extensive characterization of the cell proteome. Wide-search proteomics is a powerful tool to identify and quantify an enormous number of proteins and to tackle problems related to all areas of biology and medicine [1]. In the past few years, the study of the protein turnover rate on a large-scale has emerged as a new dimension in proteomics research, and a number of different approaches have been proposed to allow a reliable estimate of protein synthesis and degradation rates (extensively reviewed in [2]). Indeed, protein turnover is tightly regulated by several cellular processes which allow the maintenance of an efficient and functional protein pool and, at the same time, remove from the cellular environment unfolded, misfolded, degraded, non-functional or damaged proteins, as well as insoluble aggregates [3]. Hence, a dysfunctional proteostasis impacts the pathophysiology of many diseases, in particular neurodegenerative disorders but also cancer and metabolic conditions [4,5,6].

Historically, the first studies investigating the issue of protein synthesis and degradation involved the incorporation into proteins of radiolabeled amino acids and allowed the protein turnover rate to be expressed only as total protein dynamics [7]. Later on, proteomic approaches, mainly based on the exposure to labelled amino acid(s) of either cells cultured in vitro, or of small experimental animals in vivo, were used to estimate the rate of synthesis and/or degradation of individual proteins on a large-scale level [8,9,10,11]. The most widely used approach is known as dynamic-SILAC (often referred also as pulsed-SILAC or pSILAC), an evolution of the classical SILAC approach [12] in which cells are first cultured in normal unlabeled medium (the “light” medium) and then switched to a medium containing stable isotope labeled amino acids (usually ^13^C labelled Lysine and/or Arginine, generally indicated as “heavy” amino acids). Samples are collected at different time points, and the rates of incorporation of the heavy amino acids (which correspond mainly to the rate of synthesis) and of degradation of the pre-existing protein pool (corresponding to the degradation rate) are measured using liquid chromatography coupled to tandem mass spectrometry (LC-MS/MS). The ratio between the signal of the heavy peptide and the signal of the residual light peptide directly reflects protein turnover [2]. By applying this approach, the kinetics of each protein can be associated with its known function(s), thus expanding our knowledge of the relationships between protein expression, function, and turnover.

Primary cultures of cells are a powerful model to investigate several aspects of protein metabolism, among them protein turnover [13]. Cultures of human fibroblasts have been widely exploited as an in vitro system to investigate some pathophysiological mechanisms of disease, such as diabetes mellitus [14,15,16,17], particularly those associated with the development of diabetic complications [18,19,20]. Diabetic nephropathy (DN) is a leading cause of morbidity and mortality in diabetes [21]. Both genetic and environmental factors are associated with the development of diabetic nephropathy, particularly in type 1 diabetes mellitus (T1DM) [22]. As a matter of fact, genetic factors may either convey the risk of, or protect from, diabetic nephropathy [23]. Their expression profiles in skin fibroblasts from type 1 diabetic patients, could reflect genetic influences; therefore, they were removed from in vivo environmental influences [24].

Our laboratories have established and largely exploited the model of primary cultures of human skin fibroblasts as a tool to study protein expression in type 1 diabetes [20,25,26]. In this study, we describe the application of a dynamic-SILAC approach to primary human fibroblasts derived from diabetic subjects with and without diabetic nephropathy with the purpose of extensively characterizing the dynamics and the abundance of the proteins and, whenever possible, deriving meaningful information regarding the energetic factors underlying the relationship between stability and protein abundance in this cellular model.

## 2. Results

### 2.1. Dynamic-SILAC Experiment and Determination of prOtein Half-Lives

To accurately estimate protein half-life in a model of diabetic nephropathy, we applied a dynamic-SILAC approach to fibroblasts obtained from 10 type 1 diabetic patients, 5 of whom were affected by diabetic nephropathy. Cells were cultured as described in Section 4, and the dynamic-SILAC experiment was performed in steady-state conditions, when cells were at confluence. To verify that no evident morphological changes occurred during the timeframe of the experiment, we regularly checked cell morphology and counted viable cells. Results for an exemplifying subject are reported in Table 1.

**Figure 1:**
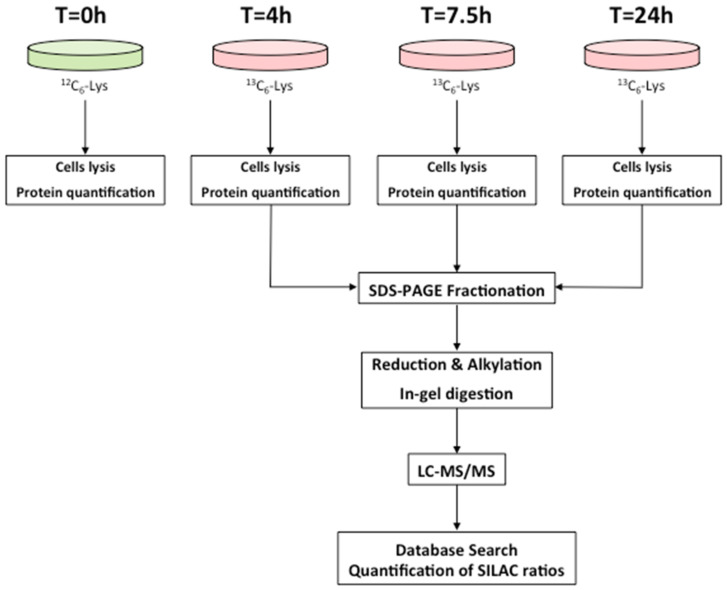
Proteomics workflow used to estimate turnover rates of individual proteins. Cells were grown in the SILAC heavy medium and collected at 4, 7.5, and 24 h. Following cell lysis and protein quantification, a fractionation step by SDS-PAGE was performed, and proteins were then digested and analyzed by LC-MS/MS as detailed in Section 4.

The proteomic workflow adopted to estimate individual protein turnover rates is described in Figure 1. On average, 1287 proteins were confidently identified and quantified in each of the 10 subjects, with a standard deviation SD equal to 86 (more details about the number of identified proteins in each sample are reported in Supplementary Table S1). As described in the experimental procedures, to increase the robustness of the estimate of the turnover constant *k*, we decided to consider only those proteins that were quantified at 24 h and at least one out of the other two time points. After filtering, a total of 1661 different proteins from the 10 subjects were considered for the parameter *k* estimate.

**Figure 2:**
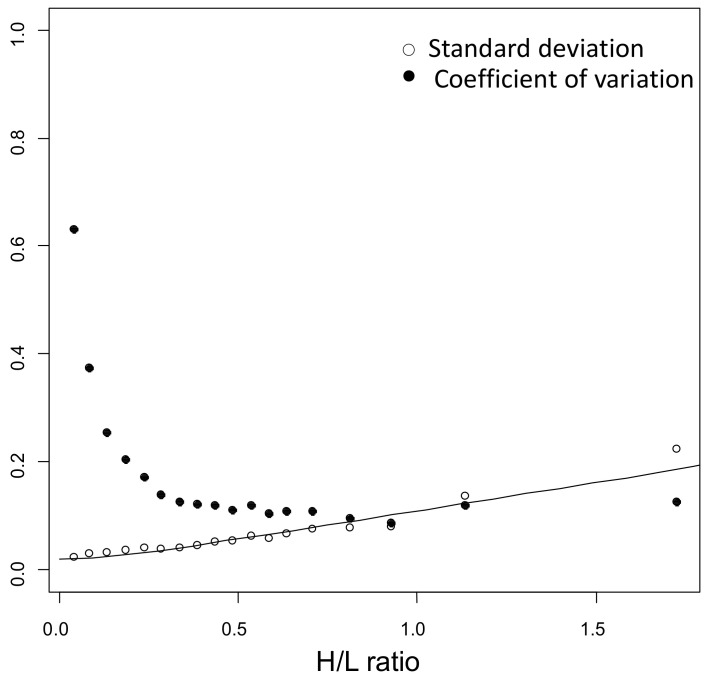
Model of technical variability. The standard deviation (SD, empty circles), and the coefficient of variation (CV, solid circles) are not constant with the average of the heavy to light ratio (H/L). Therefore, the model $\mathrm{SD}=\sqrt{\alpha^{2}+\beta^{2}\cdot r^{2}}$ was used to fit the standard deviation. The model was derived from replicated measurements of peptides.

As specified in Section 4, protein quantification was obtained by the software as the median value of the quantification of all unique peptides belonging to each protein. For these proteins, a model of technical variability was derived from peptide measurements using these latter as replicated protein measurements (see Section 4). As shown in Figure 2, neither the standard deviation (SD, empty circles) nor the coefficient of variation (CV, solid circles) are constant; the former increases, whereas the latter decreases with the average of the heavy to light ratio (H/L).

Therefore, we excluded a constant SD and constant CV model for our data and adopted the following model describing the SD as a function of H/L ratio r:

(1) $$\mathrm{SD}=\sqrt{\alpha^{2}+\beta^{2}\cdot r^{2}}$$

where α and β are constants fixed at the values of 0.02 and 0.1, respectively. The fit of the model of technical variability is shown in Figure 2.

**Figure 3:**
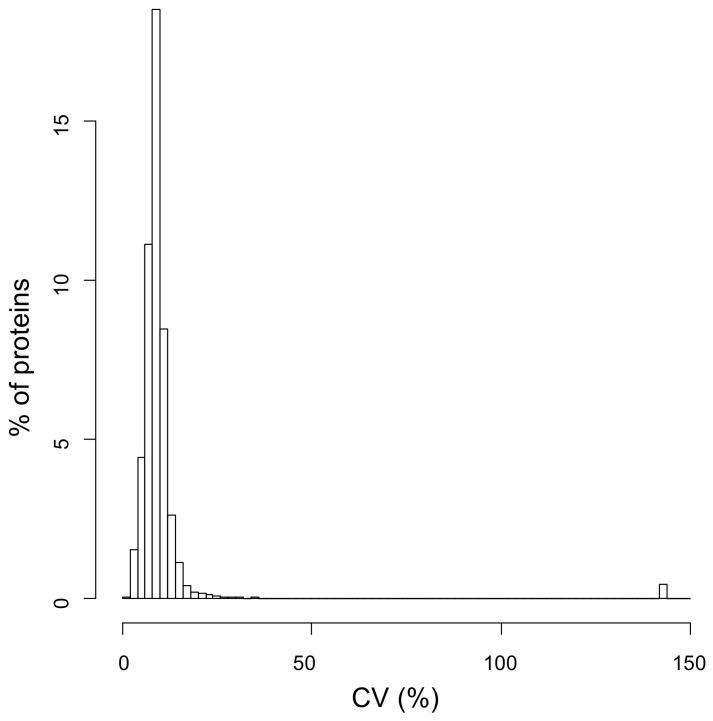
Coefficient of variation of the parameter estimate. Distribution of the CV of the parameter *k* estimate of the 1661 proteins in our dataset identified by fitting the H/L ratio across different time points.

The rate constant parameter *k* of the 1661 proteins in our dataset was identified by fitting the H/L ratio r data across time (see details in Section 4), using the least square method weighted accordingly to the model of technical variability (Equation (1)). To assess the goodness of fit, we considered the CV of the parameter estimate, whose distribution is shown in Figure 3.

From the distribution plot, it is clear that for a vast majority of proteins, the parameter *k* was estimated with very good precision, i.e., with a CV lower than 15%. A total of 1642 different proteins across all subjects (on average, 935 proteins for each subject, with a standard deviation SD equal to 119) showed a CV of the parameter estimate lower than 50% and were retained for further analysis (Table S2).

**Figure 4:**
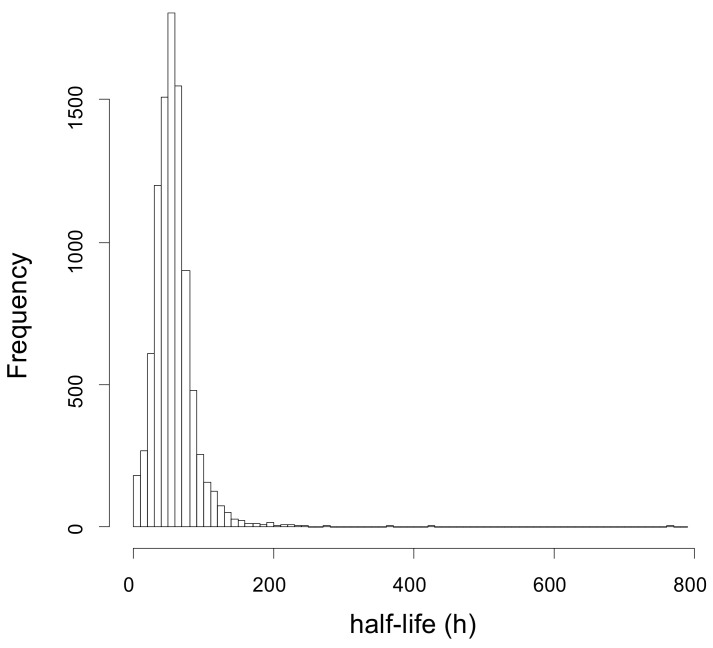
Distribution of the mean values of half-life T_1/2_. The vast majority of proteins show a half-life between 3 and 200 h with an average of 59.9 h.

Figure 4 shows the average half-life distribution of the 1642 analyzed proteins, where the half-life T_1/2_ is computed as log2/*k*. Considering only the proteins for which a reliable estimate of *k* could be obtained in at least 2 out of 10 samples (in total 1338 proteins), the distribution of the mean values of T_1/2_ ranges between 3 and 573 h, being on average equal to 59.9 h (Table S3). Note that the protein with the highest T_1/2_ is keratin 5, probably a contaminant protein that could be filtered out of the dataset. In this case, the highest T_1/2_ would be 477 h.

### 2.2. Protein Turnover in Diabetic-Controls vs. Diabetic-Nephropathic Subjects

After evaluating the normal distribution of data (Saphiro-Wilk test at 0.05 level), a *t*-test was performed on the T_1/2_ value of 974 proteins, for which *k* was estimated in at least two subjects for each group, i.e., diabetic-controls vs. diabetic-nephropathic subjects. For 20 proteins a *p*-value ≤ 0.05 was found (Table 2), although it was not significant after correction for multiple testing. Probably the high variability of the data, mainly linked to the biological variability of the human subjects, together with the low numerosity of the dataset, does not allow for significant *p*-values after correction.

Gene set enrichment analysis was also performed to assess pathways associated with the two phenotypes. The 29 Reactome pathways [27,28] were found to be significantly associated with diabetic nephropathic subjects in terms of increased protein half-life with respect to diabetic controls. These are reported in Table S4 and discussed later in the text.

### 2.3. Protein Quantification in Diabetic-Controls vs. Diabetic-Nephropathic Subjects

**Figure 5:**
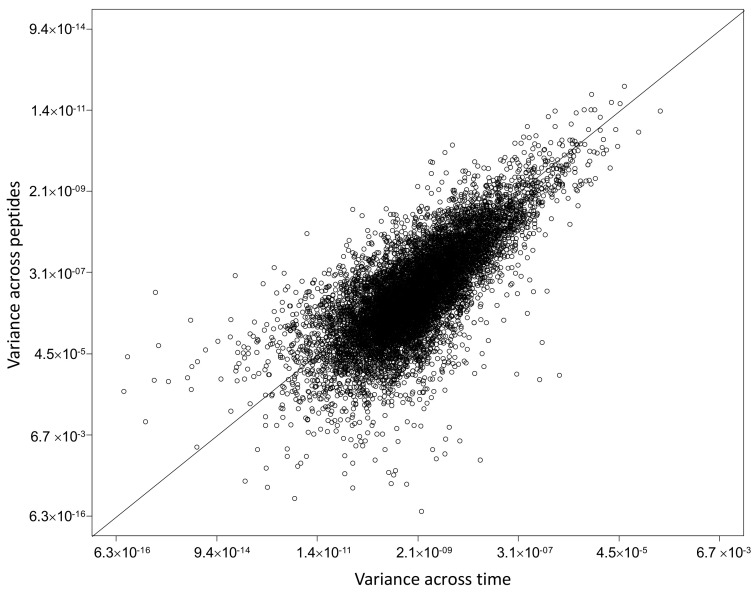
Variance across time vs. technical variance. The figure shows the variance across different time points (x axis) vs. the technical variation (y axis), measured as the variance observed across peptides matching the same protein in the same sample. The variance across time is equal to or lower than the technical variation, in line with the steady state assumption.

Protein abundance relative quantification was performed on a total of 2226 different proteins across 10 subjects (Table S5). To note that protein half-life could be reliably assessed for 1664 proteins, since for the estimate of *k* we decided to use data associated with proteins identified at least at two temporal points, one of which was set to be 24 h. This limitation does not apply for the estimate of protein abundance, which therefore led to the quantification of a higher number of proteins. Abundance levels in the three time points indirectly confirmed the steady state hypothesis. Indeed, for each protein, the variance across different time points tends to be equal to or lower than the technical variation, measured as the variance observed across peptides matching the same protein in the same sample (Figure 5). Here it is clear that protein abundance does not significantly vary across the three time points, thus indicating that a steady state can be confidently assumed.

After confirming the normal distribution of data (Saphiro-Wilk test at 0.05 level), a *t*-test was performed on 1299 proteins, for which the relative abundance was measured on at least two subjects for each group, i.e., diabetic-controls vs. diabetic-nephropathic subjects (Table S5). For 40 proteins a *p*-value ≤ 0.05 was found (Table 3), although it was not significant after correction for multiple testing.

### 2.4. Characterization of Proteins with Similar Half-Life and Abundance

Given the large range of protein half-lives spanning between 3 and 573 h (Figure 4 andTable S3), downstream analysis was performed to acquire further information on the proteins characterized by a similar range of half-lives and abundance. Considering that only a few proteins showed a significant change in protein half-life between diabetic controls and nephropathic subjects (Table 2), we decided to use the average value of *k* calculated on all 10 subjects to cluster proteins that consistently show half-life within arbitrarily predefined intervals. These groups of proteins were analyzed for their interactions and functional enrichment using STRING v. 11.0, DAVID v. 6.7, and Revigo. Results were filtered to keep only significant enriched terms with a minimum of 4 counts and a *p* value < 0.001. The results in their full form are reported in Table S6 and graphically displayed in Figure S1**.** A concise summary of this analysis is also presented in Table 4, which reports the considered half-life intervals, the number of proteins falling in each interval, and their functional characterization.

According to this analysis, the most stable proteins (with T_1/2_ > 80 h) are those involved in energy metabolism, cellular respiration, chromatin organization, and DNA packaging, while the proteins characterized by a higher turnover (i.e., those with T_1/2_ < 30 h) are mainly secreted proteins such as collagens and proteins involved in extracellular matrix organization.

**Figure 6:**
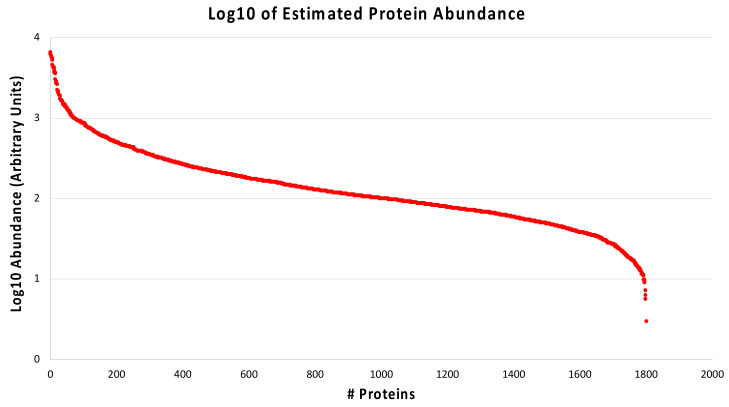
Distribution of protein abundances. The average protein abundance estimated for the entire protein set is shown in a logarithmic scale (Arbitrary Units) and spans 4 orders of magnitude.

A similar analysis was also performed using the protein abundance calculated from the overall protein expression data. The range was divided into arbitrarily pre-defined intervals to cluster proteins with similar abundance. Since only a limited number of proteins show a significantly different abundance between diabetic controls and nephropathic subjects (Table 3), we decided to average the values of all subjects, keeping only the proteins for which an estimate could be obtained for at least 2 out of 10 patients. Figure 6 shows that our estimation of protein expression data for 1801 proteins spans four orders of magnitude.

This range was divided into 8 intervals, and proteins clustered in each interval were analyzed for their interactions and functional enrichment using STRING v. 11.0, DAVID v. 6.7, and Revigo. Results were filtered to keep only significant enriched terms with a minimum of 4 counts and a *p* value < 0.001. The complete results are reported in Table S7 and graphically displayed in Figure S2. A summary of this analysis is reported in Table 5, where Log2 abundance intervals, the number of proteins falling in each interval, and their functional characterization are listed.

**Figure 7:**
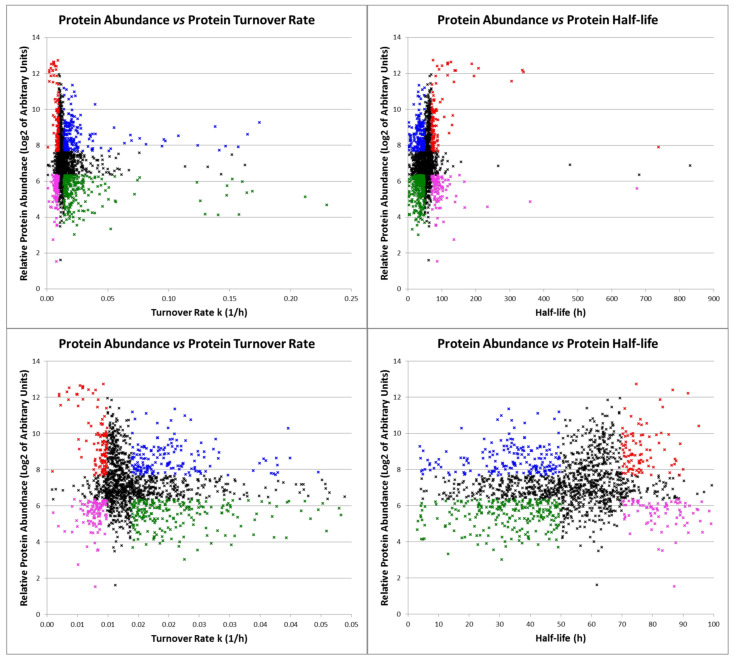
Distribution of turnover rates as a function of protein abundance. Left panels show the turnover rate constant *k* as a function of protein abundance, while right panels show the protein half-life as a function of protein abundance. Zoomed data are on display in the lower panels. Proteins characterized by a low turnover rate and high abundance are depicted in red, proteins with a high turnover rate and high abundance in blue, proteins with a high turnover rate and low abundance in green, and proteins with a low turnover rate and low abundance in pink.

In general, when plotting average protein abundance vs. average protein turnover rate *k* (Figure 7, left upper and lower panels) or, alternatively, average relative protein abundance vs. average protein half-life (Figure 7, right upper and lower panels), several clusters of proteins can be easily identified. Based on pre-defined intervals, similar to those used in the preview analyses, it is possible to distinguish four main groups of proteins: (1) proteins with low turnover rate (long half-life) and high abundance (in red in Figure 7); (2) proteins with high turnover rate (short half-life) and high abundance (in blue); (3) proteins with high turnover rate (short half-life) and low abundance (in green); and finally (4) proteins with low turnover rate (long half-life) and low abundance (in pink).

The proteins belonging to each of these groups were analyzed with STRING to highlight possible interactions and functional enrichment. The results, graphically displayed in Figure S3 and detailed in Table S8, show that the first group is particularly enriched for cytoskeletal proteins, proteins involved in chromatin organization, and proteins related to energy production. The second group involves mainly proteins related to extracellular matrix organization, cellular response to stress, metabolism of mRNA, and vesicle mediated transport. The third group is enriched for secreted proteins and proteins involved in RNA splicing, membrane trafficking, and the cell cycle. Finally, to the fourth group belong mainly proteins involved in the metabolism of proteins, hydrocarbons, and lipids.

## 3. Discussion

In this work, we quantified protein turnover using in vitro cultured fibroblast cells harvested from ten diabetic subjects, five of whom had nephropathy.

We performed a dynamic SILAC experiment on the cultured fibroblasts that had reached confluence. At time T = 0, the regular culture medium was replaced with a medium containing the heavy amino acid ^13^C_6_ Lysine, and cells were sampled at 4, 7.5, and 24 h (Figure 1). During the entire duration of the experiment, cells were regularly checked, and no significant changes in their number or morphology could be detected (Table 1). Cells were lysed, proteins were subjected to SDS-PAGE to reduce sample complexity, and in-gel digestion was performed with the protease LysC. The choice of using only labeled lysine and not also labeled arginine, as it is usually done in SILAC experiments, was made to avoid any problem that could arise from the possible arginine-to-proline metabolic conversion [29,30,31] and that could therefore affect the estimate of turnover rate. The use of LysC as a digesting enzyme yields a lower number of protein identifications with respect to the classical digestion with trypsin, since less and larger peptides are generated. However, we preferred to acquire more robust data at the expense of a smaller dataset. In total, we performed 300 nLC-MS/MS analyses. SILAC H/L ratios at different times were used to estimate the turnover parameter *k* by using weighted least squares (as detailed in Section 4). The CV of the parameter estimate, used to assess the goodness of fit, was lower than 15% in the vast majority of cases, thus confirming that the adopted model is adequate to describe the data (Figure 3). With this strategy, we were able to confidently estimate the turnover parameter *k* (and therefore the half-life) for 1642 different proteins, generating a high-quality dataset of protein turnover rate from human fibroblasts isolated from type 1 diabetic patients (Table S2).

### 3.1. Proteins with a Significantly Different Turnover in Nephropathic Subjects

On the 974 proteins for which *k* was estimated in at least two subjects for each group, we could perform a *t*-test to highlight proteins with a significant (*p* ≤ 0.05) different turnover rate between diabetic and nephropathic subjects. Only the 20 proteins listed in Table 2 turned out to have half-lives different in the two groups. By looking at the reported data, it is evident that all proteins except one (namely TOP2B) are characterized by longer half-lives in nephropathic subjects with respect to the diabetic controls. A screening of the literature reveals that almost all proteins listed in Table 2 have been reported to be implicated in nephropathy, often of diabetic origin.

#### 3.1.1. Mesangial Proteins

The Rab family of small G proteins plays important roles in mediating vesicular membrane trafficking in eukaryotic cells [32,33], and more than 60 mammalian Rab proteins have been identified and characterized.

Rab13 and its effector protein, JRAB/MICAL-L2, are involved in the transport of the cell adhesion molecules occludin and claudins to the tight junctional area in epithelial cells [34,35]. Rab13 was identified in a gene expression profiling study to be altered in a population of macrophages from nephritic NZB/W mice [36].

Knocking-down or overexpressing Rab23 affected the expression of collagen in cultured mesangial cells, thus suggesting that Rab23 may be overexpressed in FSGS mice to suppress hedgehog signaling and/or influence collagen synthesis [37]. A proteomic study conducted on mesangial cells points to the possible involvement of Rab23 in a variety of cellular events, such as gene expression, signaling, protein synthesis, organ and tissue morphology, cellular movement, and contraction function [38].

#### 3.1.2. Chaperone and Cytoskeleton Proteins

In this study, we identified TCP1 and CCT8 as proteins with an altered turnover rate in nephropathic subjects. The chaperonin-containing T-complex (TRiC/CCT complex) is a chaperone machinery that assists the folding of dozens of proteins, in particular those that appear to be slow-folding and aggregation-prone [39]. However, this complex has been known for a long time to fold actin and tubulin [40,41,42] and evidence suggests that disruptions of actin dynamics result in altered cytoskeletal organization [43]. Interestingly, TRiC/CCT was also identified in our Gene Set Enrichment Analysis as one of the major Reactome Pathways to be affected in our model of diabetic nephropathy (Table S4). It has been reported that the beta subunit of the complex may play a central role in mesangial cell hypo-contractility in diabetic nephropathy [44], while both CCT2 and CCT8 were found to be significantly altered in exosomes derived from primary human proximal tubular epithelial cells (PTEC) under diseased conditions [45].

Actin and tubulin are the major components of the cytoskeletal structure. A disassembly of the actin cytoskeleton and marked alterations of beta tubulin, a major component of microtubules, represent prominent features of DN [46,47]. Modifications of chaperone-like proteins have been previously detected in cultured fibroblasts from T1DM subjects with nephropathy, and they may be patho-physiologically related to the development of diabetic renal disease [20,48]. Changes in the cytoskeleton are key alterations in the pathophysiology of DN: substantial differences in cytoskeletal and cytoskeleton-related protein expression were found between normal subjects and T1DM patients with DN but not with T1DM patients without DN [20], suggesting that nephropathy, and not diabetes *per se*, was associated with the observed changes.

#### 3.1.3. Proteins Associated to Hydrogen Sulfide (H_2_S) Metabolism

Just like other gaseous compounds, such as nitric oxide (NO) and carbon monoxide (CO), H_2_S is known to act as a signaling molecule [49,50] and modulate a vast array of biological functions [51]. The conversion of hydrogen sulfide to thiosulfate and sulfane is catalyzed by the mitochondrial protein sulfide quinone oxidoreductase (SQRDL or SQOR) with the help of a quinone, usually ubiquinone [52].

Ubiquinone (also known as Coenzyme Q10 or CoQ10) is involved in several processes (primarily the electron transport chain) and functions as a cofactor for several enzymes, among them the SQRDL protein. CoQ10 deficiency is the cause of several human diseases, and mutations in the *COQ8B* gene result mainly in the disruption of kidney function, causing a steroid-resistant nephrotic syndrome [53]. Interestingly, H_2_S oxidation impairment causes CoQ10 associated nephrotic syndrome, a chronic kidney disease related to CoQ10 deficiency, and it has been shown that reduced SQOR levels lead to increased ROS production, thus contributing to oxidative stress in conditions of CoQ deficiency [54]. Our data show a strong increase in SQOR half-life in nephropathic subjects (123.6 h) with respect to the diabetic controls (74.6 h); however, the physiological significance of such a finding is difficult to grasp. On one hand, it may indicate a reduced enzyme efficiency and a higher oxidative stress. On the other hand, longer enzyme survival could instead determine a decrease in the H_2_S levels and a reduced oxidative stress. Therefore, the increased half-life of this enzyme in the cultured fibroblasts from T1DM subjects with nephropathy could be interpreted as an attempt to activate a protective mechanism through a reduction of oxidative stress, inflammation, mesangial cell proliferation, and an inhibition of the renin-angiotensin system activity [55,56,57].

#### 3.1.4. Proteins Involved in Translation and Kidney Hypertrophy

Kidney hypertrophy and matrix accumulation are associated with the development of long-term complications of diabetes [58], and translation has been reported to represent a potential biomarker for the prognosis of kidney disease [59].

We identified three subunits of the eIF4F complex (EIF4A1, EIF4G1, and EIF4H) and the PABPC1 protein, all of which had a turnover rate ≈20% greater in the nephropathic than in control subjects (Table 2). EIF4A is an ATP-dependent RNA helicase with low activity. However, the ATPase and helicase activities are strongly stimulated when EIF4A is in complex with eIF4G, eIF4E, eIF4B, and eIF4H [60].

Interestingly, accumulating evidence has highlighted a central role for translation in hypertrophy in models of diabetic nephropathy, both in vivo and in vitro [61,62]. Moreover, EIF4F has been reported to be a potential biomarker for membranous nephropathy prognosis [63], and PABPC1 is listed among the proteins associated with kidney diseases from the curated CTD Gene-Disease Associations dataset (http://ctdbase.org/detail.go?type=disease&acc=MESH:D007674 accessed on 1 July 2022).

#### 3.1.5. Other Proteins with Altered Turnover Rate

Glutathione S-transferase Mu 5 protein (GSTM5) exhibits an almost doubled half-life in the nephropathic vs. the non-nephropathic T1DM subjects (Table 2). Little has been reported in the literature regarding the possible association between this protein and the development or progression of diabetic nephropathy, although other members of the same protein family have been reported as putative biomarkers of diabetic nephropathy [64,65].

The half-life of Heterogeneous Nuclear Ribonucleoprotein F (HNRNPF) in nephropathic subjects is about 30% greater than that in non-nephropathic subjects (Table 2). Very interestingly, this protein is known to exert a protective role against oxidative stress and to attenuate nephropathy progression in diabetic mice and possibly in human kidneys via stimulation of Sirtuin-1 expression [66]. Therefore, the reduced turnover rate might be explained as an attempt to mitigate and counteract the adverse effects of nephropathy. Moreover, HNRNPF has been suggested to be a potential target for the treatment of hypertension and kidney injury in diabetes [67,68].

The transferrin receptor (TFRC) also shows an increased half-life in nephropathic subjects. An altered expression of TFRC has been detected on mesangial cells in IgA nephropathy [69,70], and recently the *TFRC* gene was reported to be downregulated in tubules of samples derived from patients affected by chronic kidney diseases [71].

Caprin-1 is an ubiquitous protein highly expressed in dividing cells [72]. The *Caprin-1* gene has been found to be downregulated in B2R knockout (B2R^−/−^) mice, a mouse model of diabetic nephropathy [73].

The Proliferation-associated protein 2G4 (PA2G4) and the topoisomerase DNA II beta (TOP2B) genes were found to be downregulated in the obstructive nephropathy of PAI-1–overexpressing mice [74]. PA2G4 was also evaluated as a potential biomarker in the serum of type 1 diabetes patients [75].

The ATP-Citrate Lyase (ACLY), the enzyme that converts citrate to acetyl-CoA, shows an increased half-life of about 25% in nephropathic subjects with respect to the diabetic controls. Interestingly, very recently, ACLY has been reported as a critical epigenetic regulator that promotes renal injury in obesity and type 2 diabetes [76,77], while other researchers have used two independent mouse models of kidney fibrosis to demonstrate that the AKT-dependent modulation of this enzyme is involved in kidney fibrogenesis and ECM deposition [78].

#### 3.1.6. GSEA Reveals That Proteasomal Proteins Have Longer Half-Lives in Nephropathic Subjects

Although only 20 proteins show a significant difference in protein turnover rate between diabetic controls and nephropathic subjects, GSEA highlighted a number of Reactome Pathways that are significantly different (FDR q-value < 0.05) in the two groups of patients. In particular, the proteins belonging to the pathways, identified and reported in Table S4, show an increased half-life in in all nephropathic subjects. A scrutiny of the GSEA output highlights that a large part of the proteins that contribute to the significant pathways belong to the proteasomal complex.

The ubiquitin proteasome system (UPS) plays a central role in the pathogenesis and progression of various diseases, among which is diabetic nephropathy [79,80]. The UPS is predominantly involved in protein homeostasis through the ubiquitination and proteasomal degradation of proteins. However, ubiquitination is not only involved in proteasome degradation, but also regulates the participation of substrate proteins in a variety of cell signaling pathways [80]. Proteasome inhibition has been shown to attenuate diabetic nephropathy [81] and have a protective effect against renal dysfunction [82,83]. Moreover, the deletion of the proteasome activator genes, *PA28α* and *PA28β*, resulted in protection against renal injury and retinal microvascular injury in diabetic mouse models [84]. Other researchers reported that an increased level of ROS induced by hyperglycemia covalently modifies the 20S proteasome subunits, thus decreasing its activity in the diabetic kidney [85]. Moreover, proteasome inhibitors improve renal fibrosis in rats with obstructive nephropathy [86], reduce collagen production, proliferation, and inflammation in nasal fibroblasts [87], and seem to be effective for the treatment of nephropathy [88]. Therefore, our data showing an increased half-life for a high number of proteasomal proteins in nephropathic subjects corroborates data already reported in the literature and suggests that the UPS could be a potential target for treatment of diabetic nephropathy.

### 3.2. Proteins with Different Abundance in Nephropathic Subjects

Our analysis led to the identification of 40 proteins with a significantly different abundance in diabetic controls vs. nephropathic subjects (Table 3). Most of these proteins were already reported as related to DN and are listed among the proteins associated with kidney diseases in the CTD Gene-Disease Associations dataset. For example, the GLI pathogenesis-related 2 has a fold change of -1.8 in our dataset; curiously, it has been reported that GLIPR-2 is elevated in the kidneys of patients affected by DN [89] and that miR-30e targeting GLIPR-2 is downregulated in DN, while its overexpression inhibits GLIPR-2, thus protecting from renal fibrosis in DN [90]. The fact that we found the protein to have a lower abundance in DN with respect to the diabetic controls seems, therefore, to be in contrast with the previous observations. However, it is worth noting that GLIPR-2 was found to be elevated in nephropathic kidneys with respect to normal kidneys, while we observed a reduction with respect to diabetic subjects.

We identified three members of the SLC25A family (the phosphate carrier SCL25A3, and the adenine nucleotide translocators SLC25A5 and SLCA25A6), all of them with a reduced abundance in nephropathic subjects. SCL25A3 was found to be differentially expressed in sclerosis-prone ROP-Os/+ and sclerosis resistant C57-Os/+ mouse kidneys [91], and both *SLC25A5* and *SLCA25A6* genes are reported to be modulated in type 2 diabetic patients with end-stage renal disease [92]. Interestingly, other 3 proteins (NDUFB10, SDHB and COX4I1) known to be functionally related to the SLC25 complex were identified with a lower abundance in nephropathic subjects, and with a fold change very similar to that found in the SCL25 proteins. Moreover, other three proteins that function generally in the processes of electron transport (NQO1, CYB5A, and QDPR) were also found to have decreased abundance in DN patients. The quinoid dihydropteridine reductase (QDPR) has been suggested to be an important modulator of diabetic nephropathy through the regulation of the TGF-β1/Smad3 signaling pathway [93] and to play an important role as a protective factor against oxidative stress [94], while NQO1 polymorphism has been recently associated with the risk of diabetic nephropathy [95]. Altogether, these data suggest a possible impairment of general electron transfer activity—in particular in the mitochondria—in nephropathic subjects, in agreement with data reported in the literature [96,97].

Three members of the tubulin family (namely TUBB4A, TUBB4B, and TUBA1C) show an increased abundance, in agreement with previous data that demonstrated that tubulins are strongly modulated in nephropathic subjects [20]. Moreover, other cytoskeletal proteins and cytoskeletal-regulating proteins with different abundances were identified in our study, namely SYNE1, CAPNS1, MTPN, MYO1B, and RRAS. Microarray studies have highlighted CAPNS1 as being modulated in membranous nephropathy [63] and in immunoglobulin A nephropathy [98], while other studies have reported the importance of miR-375 (for which myotrophin MTPN is a target) for glucose homeostasis and as a potential biomarker in type 2 diabetes [99,100]. Finally, both SYNE1 and MYO1B have been described as related to diabetic nephropathy before [101,102,103].

Although, to the best of our knowledge, not much is known about a possible role of the DNA repair proteins, XRCC5 and XRCC6, in the context of diabetic nephropathy. Recently an association between other members of the same protein family (XRCC1 and XRCC3) and diabetic nephropathy has been suggested [104].

We also identified two proteasomal proteins (PSMA7 and PSMB2), with some other functional related proteins (RPS3, RPS3A, and EEF1B2), all of them with a lower abundance in nephropathic subjects. The role of proteasomal proteins in the context of nephropathy was discussed above (paragraph 3.1.6). Interestingly, RPS3 was described to be associated with diabetic nephropathy [92], while RPS3A was also reported to have an increased expression in membranous nephropathic kidneys [105].

Galectin-3 (LGALS3) is upregulated under diabetic conditions, providing protection toward tissue injury induced by advanced glycation end-products (AGEs) [106]. It has been considered as a possible therapeutic target for prevention and treatment of diabetes and its complications [107]. In our analysis, LGALS3 has a reduced abundance in nephropathic subjects, suggesting a possible lack of a protective effect in this group of patients. On the other hand, we found SerpinB2 to be more abundant in nephropathic subjects. Interestingly, reduced levels of SerpinB2 have been associated with the delayed development of diabetic nephropathy [108].

Other proteins found with an altered abundance in our study and known to be related to diabetic nephropathy are CTSD, FHL2, and Sec31A. This latter was associated with DN [109] and is involved in the inhibition of nerve regeneration in diabetic neuropathy [110]. CTSD expression was found to be altered in the renal tubular epithelium in patients with DN [111], and very recently, a urinary proteomic study conducted on a large cohort of type 1 diabetic subjects identified cathepsin D as a promising biomarker of rapid eGFR (estimated glomerular filtration rate) decline, which reflects kidney injury [112]. FLH2, a protein implicated in Wnt/β-catenin signaling, plays a crucial role in albuminuria and has been indicated as a potential therapeutic target against diabetic kidney damage and fibrotic kidney disease [113,114]. Finally, we found a member of the MAPK family, namely MAPK14, with a reduced abundance in nephropathic subjects with respect to diabetic controls. In agreement with our data, the implication of MAPK signaling in the development and progression of diabetic nephropathy has been amply documented [115,116].

### 3.3. Proteins with Similar Half-Life and Abundance Are Functionally Related

The data synthetically summarized in Table 4 and fully displayed in Table S6 and Figure S1 highlight the notion that proteins with similar half-lives are also very often functionally related. To perform this analysis, proteins were divided into 9 arbitrary groups based on their estimated half-lives, and the enrichment of gene ontology terms was assessed using the bioinformatic tools specified in Section 4. What emerges from this analysis did not come entirely as a surprise, since our data reveal that the most stable proteins (i.e., those with T_1/2_ > 70 h) are mainly mitochondrial, nuclear, and cytoskeletal proteins involved in energy metabolism, cellular respiration, structural functions, protein folding, translation, chromatin organization, and DNA packaging. All these basic and very important biological functions rely on proteins that are also characterized by a medium-to-high abundance (Table 5, Table S7 and Figure S2), and therefore their rapid turnover would require a very high energy consumption. In other words, the cell invests lots of energy in the synthesis of these proteins, and therefore their half-lives are conveniently long. On the other hand, if cells need to modulate the abundance of these proteins (either increasing or decreasing it) the adjustment to the new conditions cannot be obtained in a short time, therefore requiring a longer adaptation.

Proteins characterized by shorter half-lives (between 60 and 70 h) are mainly ribosomal and proteasomal, proteins related to RNA metabolism and to Ras signal transduction. Half-lives in the range 50–60 h are typical of proteins related to cell cycle, vesicle mediated transport, and actin cytoskeletal organization, while in the range 30–50 h fall predominantly proteins involved in mRNA processing, in small GTPase mediated signal transduction and Golgi vesicle transport. Finally, it was not completely surprising to identify mainly secreted proteins and proteins involved in extracellular matrix organization among those characterized by a higher turnover rate (i.e., those with T_1/2_ < 30 h). Indeed, for the category of secreted proteins, our estimated half-life is given by the contribution of two different processes: the turnover rate and the rate of secretion. Since we only measured intracellular proteins, we cannot distinguish between these two processes, although the particularly short half-life of these proteins suggests that the rate of secretion is probably much faster than the intracellular turnover rate. Regarding this aspect, it is interesting to note that collagens have estimated half-lives that are generally shorter (although not statistically significant) in nephropathic subjects with respect to diabetic controls. This might reflect the notion that nephropathic conditions are characterized by increased matrix accumulation [58].

**Figure 8:**
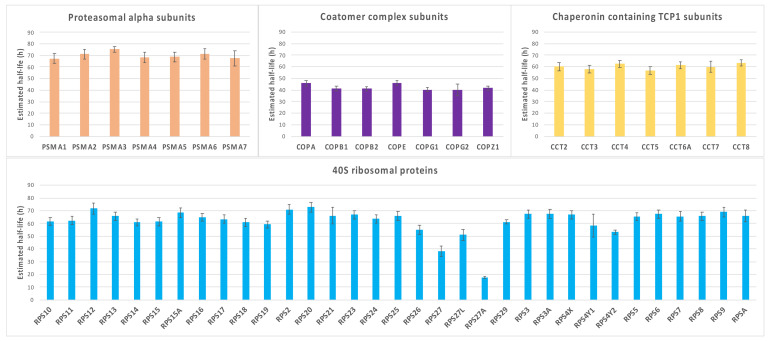
Half-lives of proteins belonging to selected macromolecular complexes. The graphs show the estimated half-lives for proteins that are part of specific, well-characterized macromolecular complexes. Error bars indicate standard errors.

To further confirm, as already reported by others [13,117], that proteins involved in common biological processes tend to have similar turnover rates, we compared the half-lives of a number of proteins that are subunits of specific, well-characterized macromolecular complexes. Some examples of this analysis are reported in Figure 8, where the half-lives of proteasomal alpha subunits, subunits of the coatomer and of the chaperone protein TCP1 complex, and ribosomal subunits of the 40S complex are shown.

**Figure 9:**
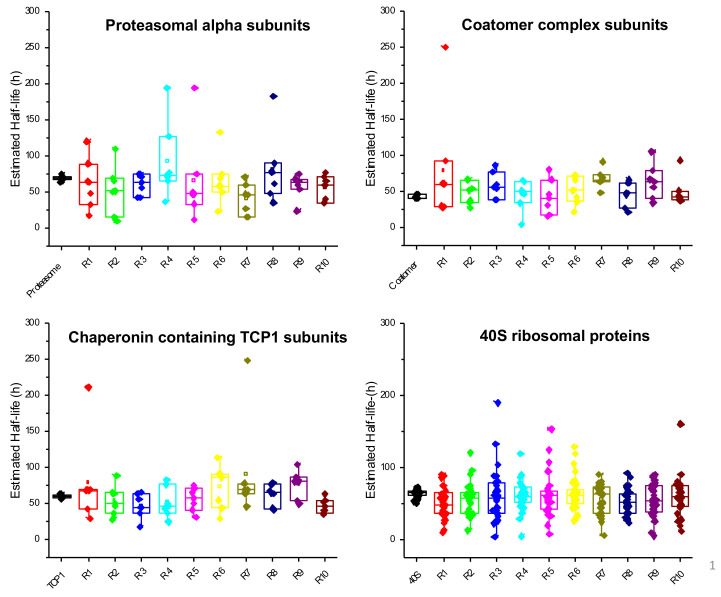
Box plots showing the distribution of half-lives for proteins belonging to the macromolecular complexes indicated in Figure 8, compared to the distribution of half-lives relative to 10 randomly selected populations of proteins generated automatically from the same dataset.

It is evident that proteins belonging to the same functional complex have very similar estimated half-lives, with the remarkable exception of proteins RPS27A and RPS27 of the 40S ribosomal complex, which show a much faster turnover rate as compared to almost all the other subunits. Interestingly, the *RPS27A* gene codes for a single copy of ubiquitin fused to the ribosomal protein S27a; therefore, it is post-translationally regulated, and its turnover rate might therefore be strongly influenced by this process. RPS27 is a ribosomal protein with extra-ribosomal functions: it has been reported as involved in DNA repair, transcription, and signal transduction [118,119], and for these unique features, it is conceivable that its turnover rate is regulated independently of the other ribosomal subunits. To rule out the possibility that the constant trend visible in Figure 8 might be due to a fortunate coincidence, we compared the distribution of half-lives of the subunits of each complex with the distribution of 10 populations randomly generated starting from the same dataset and using the same number of proteins. The selection of the populations was performed automatically using the “Random” function of Excel. As shown in Figure 9, it is evident that in all cases the pattern relative to the randomly selected proteins is much more scattered compared to the behavior of the proteins belonging to some complex, indicating that the similar turnover rate estimated for subunits belonging to the same complex reflects a true cellular regulation.

Similarly to what we did for the categorization of proteins with comparable turnover rate, we decided to group proteins based on their relative protein abundance. To this purpose, the abundance range was divided in arbitrarily pre-defined intervals, and GO-enriched terms were assessed for each cluster. Table 5 summarizes the results of this analysis (for more detailed results, see Table S6 and Figure S2), which show that the most abundant proteins are those related to DNA packaging, cytoskeletal organization, translation, RNA metabolism, and energy production. Protein folding and vesicle-mediated transport are mainly associated with proteins with an average medium/high abundance, while terms related to nucleotide metabolism, RNA splicing, cellular respiration, and the cell cycle are particularly enriched among proteins with a medium/low abundance. Finally, proteins characterized by low abundance are mainly related to the TCA cycle, mRNA and protein transport, nucleotide biosynthesis, and redox processes.

When protein half-lives are plotted as a function of abundance (Figure 7), some particularly interesting clusters of proteins emerge. Not surprisingly, proteins with both a long half-life and high abundance (in red in Figure 7) belong mainly to the category of structural proteins and proteins related to very basic and important cellular functions such as chromatin organization and energy production. Proteins with a short half-life and high abundance (in blue in Figure 7) require a particularly high energy consumption to be maintained and are mainly involved in extracellular matrix organization, cellular response to stress, metabolism of mRNA, and vesicle-mediated transport. Note that, as discussed above, extracellular matrix proteins are probably in this category (i.e., short half-life) only because we cannot distinguish between turnover rate and secretion rate. Beside proteins that are secreted, proteins with a short half-life and low abundance (in green in Figure 7) are mainly involved in RNA splicing, membrane trafficking, and the cell cycle. It is worth noting that the concentration of these proteins can be rapidly increased in case of need by simply limiting their degradation rate, leading to their fast accumulation. Finally, proteins with a long half-life and low abundance (in pink in Figure 7) are involved in the metabolism of proteins, hydrocarbons, and lipids. The cell does not require many copies of these proteins, but nevertheless they are involved in basic cellular functions and appear, therefore, particularly stable.

We must finally highlight that it is difficult to compare our results with similar data previously published by other research groups. For instance, when comparing the average protein half-lives obtained in this study across 10 subjects with those reported in the seminal work of Schwanhausser et al. on a single sample [9], it appears that the half-life that we estimate tends to be consistently lower, although the order of magnitude is pretty similar. These differences can be attributed to several reasons. First of all, we used patient-derived primary fibroblasts, whereas murine fibroblasts were used in [9], and moreover, we considered cells at confluence, whereas in Schwanhausser et al., the total protein abundance is assumed to double during the duration of one cell cycle. Another important difference is that the last time-point measured in our experiment is 24 h vs. 13.5 h in Schwanhausser et al. Given that the ratio *r* of proteins labeled with heavy and light amino acids increases slowly for high half-life proteins, having late time points in the experimental set-up should guarantee a better estimate of *k* for these kinds of proteins. Finally, while in Schwanhausser et al., a simple least square estimation is used, here a weighted least square estimate of parameter *k* was adopted.

A more reasonable comparison can be made with the data reported by Welle et al. [120], where protein turnover rate is estimated in immortalized human fibroblasts. Although both the analytical approach (classical dynamic-SILAC vs. a hyperplexing strategy) and the cellular model (patient-derived primary fibroblasts vs. immortalized fibroblasts) are not identical, the half-lives we estimated appear to be in good agreement with the data they have published (same average value of k and a correlation coefficient >0.6), thus further supporting the reliability of our dataset.

## 4. Materials and Methods

### 4.1. Patients’ Selection and Enrolment

We sought to quantify protein turnover in unperturbed fibroblast cells in a population of 10 diabetic subjects, five of whom had nephropathy. Five Caucasian T1DM patients with DN (i.e., with a urinary albumin excretion rate (AER) >200 mg/min in sterile urine, not associated with other proteinuric diseases) and five T1DM patients without DN (AER <20 mg/min) were recruited. The aims of the study were explained in detail, and each subject gave informed consent to the study. The protocol had been approved by the Ethical Committee of the Medical Faculty at the University of Padova and was performed according to the Helsinki Declaration (1983 revision).

The patients’ characteristics are reported in detail elsewhere [20]. Age (means: 36–39 yrs), male/female ratio (2/3), body mass index (BMI) (means: 22–24 kg/m^2^), diabetes duration (≈20 yrs), and glycated hemoglobin levels (means: 9–11%) were not different between the two groups of diabetic subjects. Their albumin excretion rate (AER) was determined on three timed overnight urine collections, by a turbidimetric method (Turbiquant Albumin, Dade Behring, Marburg, Germany), and the median value was used for DN classification. The mean blood pressure was calculated as diastolic blood pressure plus one-third systolic (i.e., pulse) pressure. All drugs were suspended the day before the study.

### 4.2. Cell Collection and Culture

The fibroblasts were obtained by skin biopsies as described in detail elsewhere [25]. The skin explants were incubated at 37 °C after addition of HAM’S F-10 Nutrient Mixture medium (Sigma Aldrich, St. Louis, MO, USA) supplemented with 20% foetal bovine serum (FBS Sigma Aldrich), 1 mM glutamine, (Sigma Aldrich), 100 U/mL penicillin and 100 µg/mL streptomycin (Sigma Aldrich). The growth medium was changed every 3–4 days. Usually, the fibroblasts became visible after 4–5 days of culture, and they reached the confluence after about 3 weeks. Thereafter, the culture medium was aspirated, and cells were washed three times with PBS. The fibroblasts were recovered by adding 0.05% trypsin and 0.02% EDTA (Sigma-Aldrich), transferred into 75 cm^2^ flasks and cultured with the culture medium containing 10% FBS. After the third passage, cells were frozen and kept in liquid nitrogen. Before each experiment, the fibroblasts were thawed and grown up to the 4th–5th passage.

### 4.3. Dynamic-SILAC Experiment, Sample Preparation and In-Gel Protein Digestion

For the dynamic SILAC experiment a custom-made Dulbecco’s Modified Eagles Medium (DMEM) without L-arginine, L-lysine, and L-glutamine (Athena Enzyme systems, Baltimore, MD, USA) was used after adding L-arginine, L-glutamine, and L-lysine (Sigma) or ^13^C_6_-Lysine (Cambridge Isotope Laboratories, Tewksbury, MA, USA), and 10% dialyzed foetal bovine serum (FBS, Invitrogen, Paisley, UK). To determine protein half-lives, a dynamic-SILAC approach was used. The fibroblasts were cultured in standard, light (L) DMEM medium until they reached confluence. Thereafter, the standard medium was removed, the cells were washed three times with 10 mL of phosphate-saline buffer (PBS, pH = 7.4, Sigma Aldrich), and the heavy medium (containing the^13^C_6_-Lysine) was added to the culture at time T = 0.

Cells were harvested at 4, 7.5, and 24 h, lysed by the addition of 70 μL of Tris-HCl 62.5 mM, pH 7.2, 1% SDS, and protease inhibitors (Protease Inhibitor Cocktails, Sigma Aldrich), and by repeated freeze-thaw cycles in liquid nitrogen. The samples were then centrifuged at 14,000 rpm for 15 min to remove cell debris, and the protein concentration in the supernatant was quantified by the Lowry method. Thereafter, 70 μg of total proteins for each time point and for each subject were loaded onto a 12% precast gel (NuPAGE, Invitrogen). The electrophoretic separation was performed by applying a constant voltage of 80 V for 30 min. Gel was then stained for 3 h with colloidal coomassie (SimplyBlue Safe Stain, Invitrogen) and then destained with water. Each gel lane was cut into five bands that were then subjected to in-gel enzymatic digestion with LysC protease (Promega, Madison, WI, USA) as described in [121].

### 4.4. LC-MS/MS Analysis

Each of the fractions obtained as specified above was analyzed by LC-MS/MS. Data were submitted for database search and quantification of SILAC H/L ratios. The analysis was conducted with a LTQ-Orbitrap XL mass spectrometer (Thermo Fisher Scientific) interfaced to a nano-HPLC Ultimate 3000 (Dionex—Thermo Fisher Scientific, Waltham, MA, USA). Samples were loaded into a 10 cm pico-frit capillary column (75 μm I.D., 15 μm tip, New Objective, Littleton, MA, USA) packed in-house with C18 material (Aeris peptide 3.6 um XB-C18, Phenomenex, Torrance, CA, USA), and peptides were separated by a linear gradient from 3% to 40% acetonitrile/0.1% formic acid in 40 min. The instrument operated in a Top4 data-dependent mode, with a full MS scan from 300 to 1700 Da acquired at high resolution (60,000) in the Orbitrap, followed by 4 MS/MS spectra of the most intense ions acquired in the linear trap.

To increase the number of identifications and robustness of quantification, each sample was analyzed twice. After the first round of analysis, all data files were searched against the human section of the Uniprot database (as specified below). All peptides that were identified with high confidence were used to create a static exclusion list that was then inserted into the instrument method. All samples were analyzed again under identical chromatographic and instrumental conditions, but with the application of the static exclusion list. The second round of analysis allows for an increase in the number of protein groups and unique peptides confidently identified, thus increasing the robustness of quantification. A series of representative Venn diagrams showing the performance of this analytical strategy are reported in Figure S4. In total, 300 LC-MS/MS analyses were performed (2 analyses for each of the five gel bands, for the three time points, and for the 10 patients).

### 4.5. Protein Identification and Quantification

All raw files generated in the study were analyzed with the software Proteome Discoverer (version 1.2, Thermo Fisher Scientific) interfaced to a Mascot server (version 2.2.4, Matrix Science, Chicago, IL, USA). The search was performed against the human section of the Uniprot Database (www.uniprot.org, accessed on 1 April 2013) using the MudPIT protocol. LysC was selected as the digesting enzyme, with up to one missed cleavage allowed. Precursor and fragment tolerances were set at 10 ppm and 0.6 Da, respectively. Carbamidomethyl cysteine was selected as a static modification, while methionine oxidation and ^13^C_6_-lysine were set as variable modifications. Data were filtered based on the search against a corresponding randomized database, and the false discovery rate (FDR) was calculated by the software. Only proteins identified with at least 2 unique peptides with high confidence (>99%) were considered positive hits. SILAC ratios were calculated by the software for each identified peptide, and peptides were grouped into protein families according to the principle of maximum parsimony. Protein quantification was calculated as the median value of the quantification of all peptides belonging to the same protein family. For each cell line, all the data obtained from the five gel bands, both with and without the application of the excluding list, were merged into a single msf output file. Msf files relative to the three time points for each cell line were finally merged into a single multi-report file.

### 4.6. Kinetic Analyses

As derived in the following equations, for constant incorporation rates, the logarithm of the SILAC H/L ratios increases linearly with time; therefore, protein half-lives can be obtained by properly fitting H/L rations measured at different time points. Five biological replicates were available for each group to assess statistical significance.

Proteins labeled with light amino acids (*P_L_*) are assumed to decay exponentially with the degradation rate constant *k* (Equation (2)).

(2) $$P_{L}=P_{TOT}\cdot e^{-kt}$$

Our experiment is conducted with cells at confluence and at constant volume. Under the hypothesis of steady state, i.e., *P_TOT_*, *k* and protein synthesis constant in time, no amino acid recycling, and assuming a mono-compartmental model, the protein labelled with heavy amino acids (*P_H_*) can be expressed as the difference between the total number of a specific protein (*P_TOT_*) and *P_L_* as in Equation (3).

(3) $$P_{H}=P_{TOT}-P_{L}=P_{TOT}\cdot \left(1-e^{-kt}\right)$$

The rate constant of the protein decay *k* can then be obtained by fitting the model of the ratio r of protein labeled with heavy and light amino acids at different time points (Equation (4)).

(4) $$r=\frac{P_{H}}{P_{L}}=\frac{1-e^{-kt}}{e^{-kt}}\;$$

By taking the natural logarithm (ln), Equation (4) can be transformed into:

(5) $$\ln\left(r+1\right)=kt$$

Proteins whose SILAC ratio was not available for the time of 24 h, and for at least one of the other time points (4 or 7.5 h), were filtered out. For the remaining proteins, the parameter *k* was identified by fitting the H/L ratio r data according to Equation (5), using the weighted least square method. Weights were calculated as the inverse of the variance of ln(r + 1) data, starting from Equation (1) and using the error propagation rules.

Equation (1) is a model of the technical variability of r derived from peptide measurements, using these latter as replicate protein measurements. In more detail, the standard deviation (SD) and the coefficient of variation (CV) of the H/L ratio r were calculated for each protein, for each subject, and for each time point. Measurements with CV% higher than 50% were excluded from downstream analysis. The range of r values were then divided into intervals of the same bin size (0.05) or containing at least 10 protein measurements, and, for each interval, the median of the SDs and the CVs was considered to fit a model of technical variability.

The goodness of fit was evaluated using the precision of parameter *k* estimates; parameters with coefficients of variation higher than 50% were considered unreliable.

Once *k* is determined, the protein half-life, i.e., the time required for the amount of the protein to fall to half its initial value if the synthesis is zero, can be calculated as:

(6) $$T_{1/2}=\frac{\ln\left(2\right)}{k}$$

### 4.7. Protein Abundance

In parallel, we quantified the relative protein abundance using the sum of peak intensities of all peptides matching a specific protein divided by the number of observed peptides for that protein and by the total sum of peaks in each LC-MS/MS run [9]. Under the hypothesis of steady state, protein abundance levels were averaged across the three time points.

### 4.8. Bioinformatic and Statistical Analysis

A number of bioinformatic tools were used to assess whether proteins characterized by similar half-lives or abundance tend to share interacting partners and be associated with similar Gene Ontology (GO) terms. For this purpose, our datasets were analyzed with STRING v. 11.0 [122] to highlight physical/functional interactions among proteins and with David Bioinformatic Resources v. 6.7 [123,124] and Revigo [125] to underline and graphically visualize enriched GO terms associated with the different classes of proteins.

Gene set enrichment analysis (GSEA) was also performed on our data using MSigDB Canonical pathways gene set collection [126]. The GSEA was used to determine whether the members of a given gene set were associated with a group. If a gene set had a positive enrichment score, a significant number of its gene members had higher expression in one of the predefined groups, and the gene set was termed “enriched”. A 1000 random sample permutations were carried out, and the significance threshold was set at FDR < 0.05. All comparisons between groups were performed using the two-tailed Student *t*-test for unpaired data.

## 5. Conclusions

In this manuscript, we describe the application of a dynamic-SILAC approach to study the turnover rate and the relative abundance of proteins using a cellular model of diabetic nephropathy. Under the hypothesis of steady state, no amino acid recycling, and assuming a mono-compartmental model, we adopted a model describing the SD as a function of heavy to light ratio and estimated the parameter *k* using the least square method weighted accordingly to the model of technical variability. We could reliably estimate the turnover rate for more than 1600 proteins and the relative abundance for more than 2200 individual proteins. Several of these turned out to be significantly different in either half-life or abundance between nephropathic subjects and diabetic controls. Many of these proteins were already known to be related to diabetic complications and therefore represent possible biomarkers or therapeutic targets. However, beside the aspects strictly related to the pathological condition, the data collected in this study represent a reliable compendium of protein half-lives in human fibroblasts and a rich source of important information related to basic cell biology.

## Figures and Tables

**Table 1:** Number of viable cells repeatedly counted during the experiment.

Time Point	Average Cell Number
1 h	5.26 × 10^6^
2 h	5.68 × 10^6^
4 h	5.66 × 10^6^
7.5 h	5.56 × 10^6^
24 h	5.98 × 10^6^

**Table 2:** Proteins with a significantly different half-life in Diabetic and Nephropathic subjects.

Gene Name	Protein Description	Average Half-Lives in h (SD)		*p*-Value	Valid Values	
		Diabetic	Nephropathic		Diabetic	Nephropathic
RAB13	RAB13, member RAS oncogene family	26.4 (1.5)	32.6 (0.1)	1.90 × 10^−3^	5	2
RAB23	RAB23, member RAS oncogene family	33.0 (2.8)	42.9 (4.3)	1.10 × 10^−2^	5	3
SQRDL	sulfide quinone reductase-like (yeast)	74.6 (12.7)	123.6 (16.5)	1.54 × 10^−2^	4	2
PABPC1	poly(A) binding protein, cytoplasmic 1	41.2 (3.9)	49.1 (4.4)	1.85 × 10^−2^	5	5
IPO7	importin 7	28.7 (1.8)	34.7 (3.9)	2.02 × 10^−2^	4	5
NPM1	nucleophosmin (nucleolar phosphoprotein B23, numatrin)	48.2 (3.4)	55.6 (4.5)	2.10 × 10^−2^	5	5
TCP1	t-complex 1	58.0 (6.0)	73.7 (12.1)	2.71 × 10^−2^	5	5
EIF4A1	eukaryotic translation initiation factor 4A1	29.8 (1.8)	34.7 (4.0)	3.31 × 10^−2^	5	5
CCT8	chaperonin containing TCP1, subunit 8 (theta)	57.8 (5.2)	68.9 (9.3)	3.34 × 10^−2^	5	5
EIF4G1	eukaryotic translation initiation factor 4 gamma, 1	26.8 (1.3)	32.2 (3.8)	3.66 × 10^−2^	4	4
TFRC	transferrin receptor	23.3 (2.9)	29.2 (3.2)	3.72 × 10^−2^	4	5
TOP2B	topoisomerase (DNA) II beta 180kDa	33.0 (3.3)	25.9 (0.3)	3.93 × 10^−2^	3	2
ACTB	actin, beta	65.3 (5.3)	89.8 (20.9)	4.12 × 10^−2^	5	4
ACLY	ATP citrate lyase	35.2 (4.7)	43.5 (4.9)	4.25 × 10^−2^	5	5
ST13P4	Suppression of tumorigenicity 13 pseudogene 4	21.8 (2.5)	37.6 (7.3)	4.36 × 10^−2^	3	2
GSTM5	glutathione S-transferase mu 5	53.5 (5.9)	104.9 (18.5)	4.38 × 10^−2^	2	2
HNRNPF	heterogeneous nuclear ribonucleoprotein F	29.8 (4.0)	38.1 (6.0)	4.80 × 10^−2^	5	4
PA2G4	proliferation-associated 2G4, 38kDa	49.4 (3.7)	62.2 (12.5)	4.85 × 10^−2^	5	5
CAPRIN1	cell cycle associated protein 1	14.7 (0.9)	16.2 (1.1)	4.89 × 10^−2^	5	5
EIF4H	eukaryotic translation initiation factor 4H	28.9 (1.9)	37.6 (3.6)	4.90 × 10^−2^	2	3

**Table 3:** Proteins with a significantly different abundance in Diabetic and Nephropathic subjects.

Gene Name	Protein Description	Fold Change (Nephropathic vs. Diabetic)	*p*-Value	Valid Values	
				Diabetic	Nephropathic
GLIPR2	GLI pathogenesis-related 2	−1.8	3.30 × 10^−3^	4	4
RPS3A	ribosomal protein S3A	−1.6	3.90 × 10^−3^	5	5
TRIM25	tripartite motif containing 25	−1.5	6.41 × 10^−3^	3	4
SLC25A6	solute carrier family 25 (mitochondrial carrier; adenine nucleotide translocator), member 6	−1.7	6.95 × 10^−3^	5	5
TUBB4A	tubulin, beta 4A class IVa	2.1	9.41 × 10^−3^	5	5
NDUFB10	NADH dehydrogenase (ubiquinone) 1 beta subcomplex, 10, 22kDa	−1.7	1.21 × 10^−2^	5	4
NPC2	Niemann-Pick disease, type C2	2.5	1.47 × 10^−2^	2	4
SYNE1	spectrin repeat containing, nuclear envelope 1	−2.2	1.47 × 10^−2^	5	5
SEC31A	SEC31 homolog A (S. cerevisiae)	1.6	1.55 × 10^−2^	3	4
TUBB4B	tubulin, beta 4B class IVb	3.3	1.70 × 10^−2^	2	4
SLC25A3	solute carrier family 25 (mitochondrial carrier; phosphate carrier), member 3	−1.7	2.40 × 10^−2^	5	5
DNAJC8	DnaJ (Hsp40) homolog, subfamily C, member 8	−1.8	2.40 × 10^−2^	5	4
COX4I1	cytochrome c oxidase subunit IV isoform 1	−1.7	2.42 × 10^−2^	5	5
LGALS3	lectin, galactoside-binding, soluble, 3	−1.4	2.57 × 10^−2^	5	5
NQO1	NAD(P)H dehydrogenase, quinone 1	−2.1	2.64 × 10^−2^	5	5
SERPINB2	serpin peptidase inhibitor, clade B (ovalbumin), member 2	1.6	2.76 × 10^−2^	2	4
XRCC6	X-ray repair complementing defective repair in Chinese hamster cells 6	−1.4	2.80 × 10^−2^	5	5
SDHB	succinate dehydrogenase complex, subunit B, iron sulfur (Ip)	−1.6	2.85 × 10^−2^	4	5
FHL2	four and a half LIM domains 2	−1.6	2.87 × 10^−2^	4	4
RBMX	RNA binding motif protein, X-linked	−1.6	3.03 × 10^−2^	2	3
PSMA7	proteasome (prosome, macropain) subunit, alpha type, 7	−2.1	3.18 × 10^−2^	3	3
XRCC5	X-ray repair complementing defective repair in Chinese hamster cells 5 (double-strand-break rejoining)	−1.4	3.21 × 10^−2^	5	5
SLC25A5	solute carrier family 25 (mitochondrial carrier; adenine nucleotide translocator), member 5	−1.9	3.34 × 10^−2^	5	5
RNH1	ribonuclease/angiogenin inhibitor 1	1.4	3.43 × 10^−2^	5	5
TUBA1C	tubulin, alpha 1c	1.7	3.57 × 10^−2^	2	5
CAPNS1	calpain, small subunit 1	−1.8	3.71 × 10^−2^	4	5
RRAS	related RAS viral (r-ras) oncogene homolog	−1.7	3.82 × 10^−2^	5	5
CYB5A	cytochrome b5 type A (microsomal)	−1.8	3.90 × 10^−2^	4	5
CTSD	cathepsin D	−1.4	3.95 × 10^−2^	5	5
EEF1B2	eukaryotic translation elongation factor 1 beta 2	−1.6	4.08 × 10^−2^	5	5
MAPK14	mitogen-activated protein kinase 14	−1.6	4.09 × 10^−2^	2	3
PSMB2	proteasome (prosome, macropain) subunit, beta type, 2	−1.6	4.22 × 10^−2^	2	3
MTPN	myotrophin	−1.6	4.30 × 10^−2^	5	5
MYO1B	myosin IB	1.6	4.35 × 10^−2^	5	5
APPL2	adaptor protein, phosphotyrosine interaction, PH domain and leucine zipper containing 2	−1.3	4.51 × 10^−2^	5	5
RPS3	ribosomal protein S3	−1.7	4.55 × 10^−2^	5	5
FKBP7	FK506 binding protein 7	−1.7	4.59 × 10^−2^	5	5
QDPR	quinoid dihydropteridine reductase	−2.8	4.72 × 10^−2^	4	3
HM13	histocompatibility (minor) 13	−2.0	4.74 × 10^−2^	2	4
IMPA1	inositol(myo)-1(or 4)-monophosphatase 1	−2.6	4.99 × 10^−2^	3	3

**Table 4:** Gene Ontology annotation of proteins grouped according to their average half-life. Proteins are grouped in 9 arbitrary ranges of half-life, and the most enriched and significant GO terms (Biological Process, Cellular Component, Molecular Function, and KEGG pathways) are listed for each group.

T_1/2_ Range (h)	# of Proteins	Biological Processes	Cellular Components	Molecular Functions	KEGG Pathways
<20	64	Extracellular matrix organization; collagen metabolism; cell adhesion and motility	Extracellular matrix; vesicle; collagen trimer	Extracellular matrix structural constituent; Receptor binding; Protein binding	ECM-receptor interaction; Focal adhesion
20–30	82	Small GTPase mediated signal transduction; mRNA splicing; localization	Cytoplasm; Spliceosomal complex	RNA binding; GTPase activity	
30–40	157	Intracellular transport; Translation; RNA splicing; localization	Spliceosomal complex; Nuclear part; Cytoplasmic part; Ribonucleoprotein complex; EIF3 and EIF4F	Protein binding; RNA binding; Nucleotide binding; Nucleoside-triphosphatase activity	Spliceosome
40–50	193	Protein transport; Golgi vesicle transport; RNA splicing; Translation	Cytoplasm; Vesicle; Golgi; Cytoskeleton; EIF3; Ribonucleoprotein complex; Plasma membrane part	Protein binding; Nucleotide binding; RNA binding; actin binding; GTPase activity; Translation initiation factor activity	Endocytosis
50–60	227	Vesicle mediated transport; Cell cycle progress; Membrane and cytoskeleton organization; Translation elongation; Protein folding	Cytoplasm; Ribosome; Cytoskeleton; Endoplasmic reticulum;	Protein binding; Nucleotide binding; RNA binding; GTPase activity; Structural constituent of ribosome	Aminoacyl-tRNA biosynthesis; Ribosome
60–70	272	RNA processing; Translation; Protein metabolism; Ribosome biogenesis; Protein folding; Ras protein signal transduction	Cytosol; Mitochondria; Nuclear part; Ribosome; Cytoskeleton; Proteasome complex	Structural constituent of ribosome; actin and cytoskeletal protein binding; RNA binding; Protein binding; Threonine-type endopeptidase activity.	Ribosome; Proteasome
70–80	170	Carbohydrate and protein metabolism; Oxidation-reduction process; Cellular respiration; Protein folding; Translation elongation; Acetyl-CoA metabolism;	Cytoplasm; Mitochondrion; Cytoskeleton; Endoplasmic reticulum part; Proteasome complex; Melanosome	Peroxiredoxin activity; Oxidoreductase activity; endopeptidase activity; Isomerase activity; Cytoskeletal protein binding; Coenzyme binding;	Glycolysis/Gluconeogenesis; Proteasome; Pyruvate metabolism; TCA cycle;
80–90	76	Carbohydrate metabolism; Generation of energy; Carboxylic acid, alcohol, and ketone metabolism;	Cytoplasm; Mitochondrion;	Catalytic activity; Monosaccharide binding; Oxidoreductase activity; Isomerase activity	Glycolysis/Gluconeogenesis
>90	97	Generation of energy; Oxidative phosphorylation; Carboxylic acid, and ketone metabolism; Chromatin organization and DNA packaging	Cytoplasm; ATP synthase complex; Mitochondrion; Nucleosome; Nuclear membrane; Protein-DNA complex		

**Table 5:** Gene Ontology annotation of proteins grouped according to their average abundance. Proteins are grouped in 8 arbitrary ranges of abundance (expressed as Log2 of arbitrary units), and the most enriched and significant GO terms (Biological Process, Cellular Component, Molecular Function, and KEGG pathways) are listed for each group.

Relative Abundance (Log2 A.U.)	# of Proteins	Biological Processes	Cellular Component	Molecular Function	KEGG Pathways
>9.0	202	Translation elongation; Protein folding; DNA packaging; Cytoskeleton organization; Cell redox homeostasis, Glycolytic process	Cytosol; Nucleus; Protein-DNA complex; Cytoskeleton; Vesicle; Large ribosomal subunit	RNA binding; Structural constituent of ribosome; GTP binding; Protein binding; Cytoskeletal and actin binding	Ribosome; Systemic lupus erythematosus; Pathogenic *Escherichia coli* infection
8.0–9.0	224	Translational elongation; RNA splicing; Cytoskeleton organization; Protein transport; Small GTPase mediated signal transduction; Generation of precursor metabolites and energy	Cytoplasm; Actin cytoskeleton; Small ribosomal subunit; Spliceosomal complex; Vesicle	Protein binding; GTP binding; RNA binding; Cytoskeletal protein and actin binding; Structural constituent of ribosome; Hydrogen ion transmembrane transporter activity	Ribosome; Spliceosome; Parkinson’s disease
7.5–8.0	180	Intracellular transport; Vesicle mediated transport; Localization; Vesicle and membrane organization; Small GTPase mediated signal transduction	Cytoplasm; Vesicle; Endoplasmic reticulum; Cytoskeleton; Arp2/3 protein complex; Proteasome core complex; Ribonucleoprotein complex	Protein binding; Actin binding; RNA binding; GTP binding; GTPase activity; Threonine-type peptidase activity	Proteasome; Pathogenic *Escherichia coli* infection
7.0–7.5	220	Intracellular transport; RNA processing and splicing; Translation; Membrane organization; Protein folding; Energy derivation by oxidation of organic compounds	Cytosol; Endoplasmic reticulum; Vesicles; Endomembrane system; Spliceosomal complex; EIF3 complex	Protein binding; RNA binding; Translation initiation factory activity;	
6.5–7.0	283	Nucleotide Metabolism; Heterocycle metabolism; Protein metabolism; Protein folding; RNA splicing; Redox processes; Cellular respiration; Response to oxidative stress	Cytoplasm; Intracellular organelle part; Macromolecular complex	RNA binding; Nucleotide binding; NADH dehydrogenase activity; Oxidoreductase activity; Protein binding; Cofactor binding	Aminoacyl-tRNA biosynthesis; Proteasome; Oxidative phosphorylation; Huntington’s disease; Amino sugar and nucleotide sugar metabolism; Alzheimer’s disease
6.0–6.5	261	Protein transport; Protein metabolism; Carboxylic acid metabolism; Amine metabolism; Mitotic cell cycle; regulation of ligase activity	Cytoplasm; Mitochondrion; Proteasome complex; Ribonucleoprotein complex; Organelle membrane	Protein binding; RNA binding; Nucleotide binding; Cytoskeletal proteins and actin binding; Translation initiation factor activity	Proteasome
5.0–6.0	298	Acetyl-CoA metabolism; Heterocycle metabolism; Nucleotide biosynthesis; carboxylic acid metabolism; Cellular respiration; Protein transport	Cytoplasm; Mitochondrion; Golgi apparatus part; Envelope	Catalytic activity; Hydrolase activity; Protein binding; Purine nucleotide binding; Pyrophosphatase activity	TCA cycle
<5.0	135	mRNA transport; Protein transport; Cellular localization; Oxidation-reduction process	Cytoplasm; Nuclear part; Envelope	Purine nucleotide binding; Catalytic activity; Electron carrier activity	Valine, leucine, and isoleucine degradation

## Data Availability

The mass spectrometry proteomics data have been deposited to the ProteomeXchange Consortium via the PRIDE partner repository with the dataset identifier PXD038541.

## Supplementary Materials

The following supporting information can be downloaded at: https://www.mdpi.com/article/10.3390/ijms24032811/s1.
