# Supplementary material for: Modeling SILAC Data to Assess Protein Turnover in a Cellular Model of Diabetic Nephropathy
Source: Int J Mol Sci. 2023 Feb 1;24(3):2811. doi: 10.3390/ijms24032811 (PMC9917874; doi:10.3390/ijms24032811)
Supplement: Supplementary file 1 [file ijms-24-02811-s001.zip › Supplementary material/Table S1_Revised.pdf]

Proteins identified in each sample (turnover rate)

	Diab1	Diab2	Diab3	Diab4	Diab5	Nefro1	Nefro2	Nefro3	Nefro4	Nefro5
Diab1	628	415	554	354	446	395	446	436	356	470
Diab2		679	586	407	496	426	541	502	430	506
Diab3			993	494	638	564	639	655	531	651
Diab4				566	447	377	430	425	403	453
Diab5					729	468	525	557	450	585
Nefro1						654	481	459	399	486
Nefro2							754	513	470	562
Nefro3								721	463	553
Nefro4									598	465
Nefro5										745

Proteins identified in each sample (abundance)

	Diab1	Diab2	Diab3	Diab4	Diab5	Nefro1	Nefro2	Nefro3	Nefro4	Nefro5
Diab1	866	623	674	556	618	523	559	614	538	641
Diab2		963	800	645	710	608	677	703	648	717
Diab3			1129	701	792	683	737	842	723	796
Diab4				844	670	565	583	630	629	669
Diab5					973	646	665	741	668	772
Nefro1						849	595	600	567	639
Nefro2							921	645	625	693
Nefro3								991	627	734
Nefro4									856	665
Nefro5										1000
