# Supplementary material for: Modeling SILAC Data to Assess Protein Turnover in a Cellular Model of Diabetic Nephropathy
Source: Int J Mol Sci. 2023 Feb 1;24(3):2811. doi: 10.3390/ijms24032811 (PMC9917874; doi:10.3390/ijms24032811)
Supplement: Supplementary file 1 [file ijms-24-02811-s001.zip › Supplementary material/Figure S1.pdf]

$T_{1/2} < 20$ h

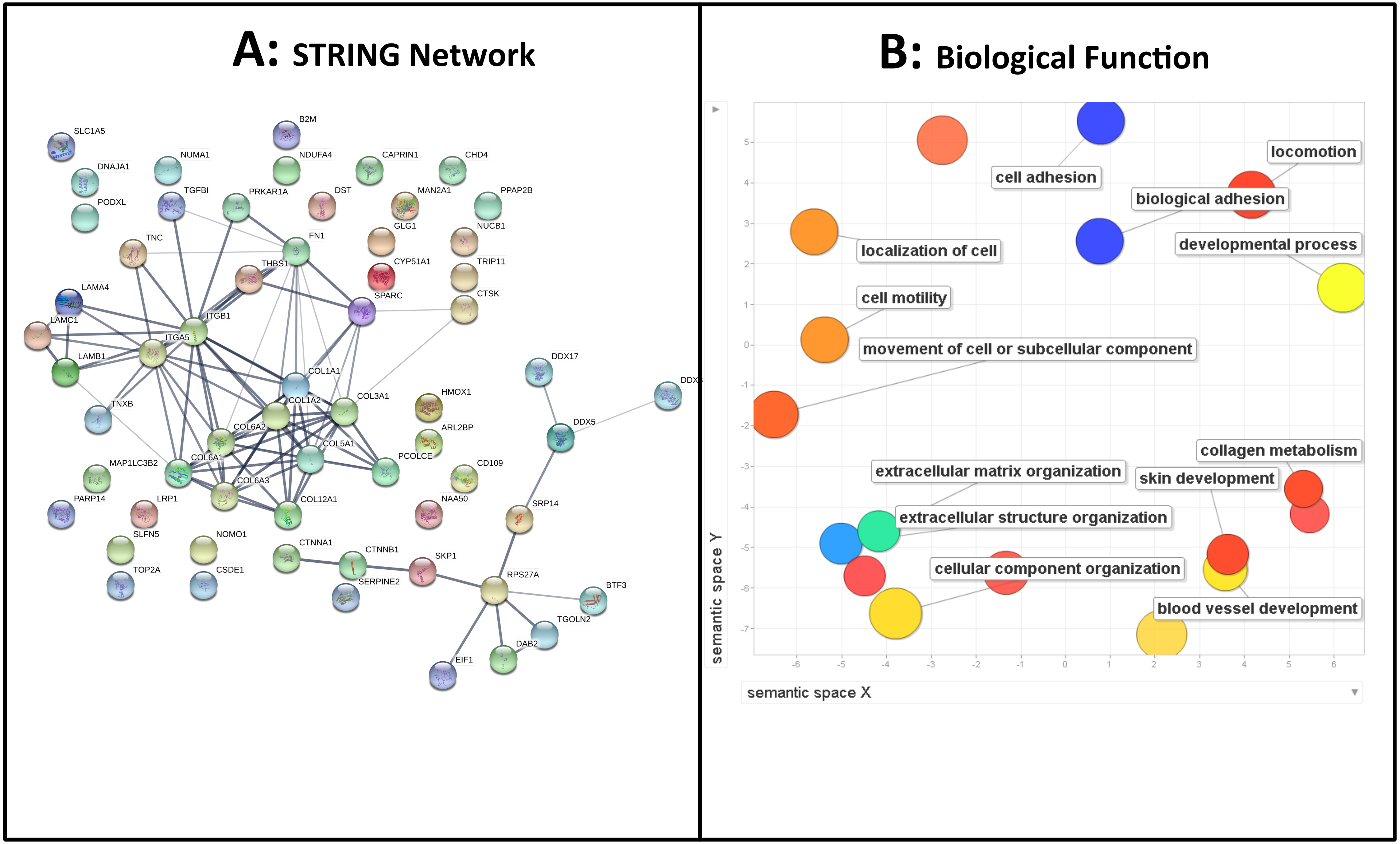
A: STRING Network
SLC1A5
B2M
NUMA1
NDUFA4
CAPRIN1
CHD4
DNAJA1
TGFBI
PRKAR1A
DST
MAN2A1
PPAP2B
PODXL
GLG1
NUCB1
TNC
FN1
THBS1
CYP51A1
TRIP11
LAMA4
SPARC
CTSK
ITGB1
LAMC1
ITGA5
LAMB1
DDX17
COL1A1
TNXB
COL1A2
COL3A1
HMOX1
DDX5
COL6A2
ARL2BP
COL5A1
PCOLCE
MAP1LC3B2
COL6A1
CD109
COL6A3
PARP14
LRP1
COL12A1
NAA50
SRP14
SLFN5
NOMO1
CTNNA1
CTNNB1
SKP1
TOP2A
CSDE1
SERPINE2
RPS27A
BTF3
TGOLN2
DAB2
EIF1
B: Biological Function
cell adhesion
locomotion
biological adhesion
localization of cell
developmental process
cell motility
movement of cell or subcellular component
collagen metabolism
extracellular matrix organization
skin development
extracellular structure organization
cellular component organization
blood vessel development
semantic space Y
semantic space X

20 h ≤ $T_{1/2}$ < 30 h

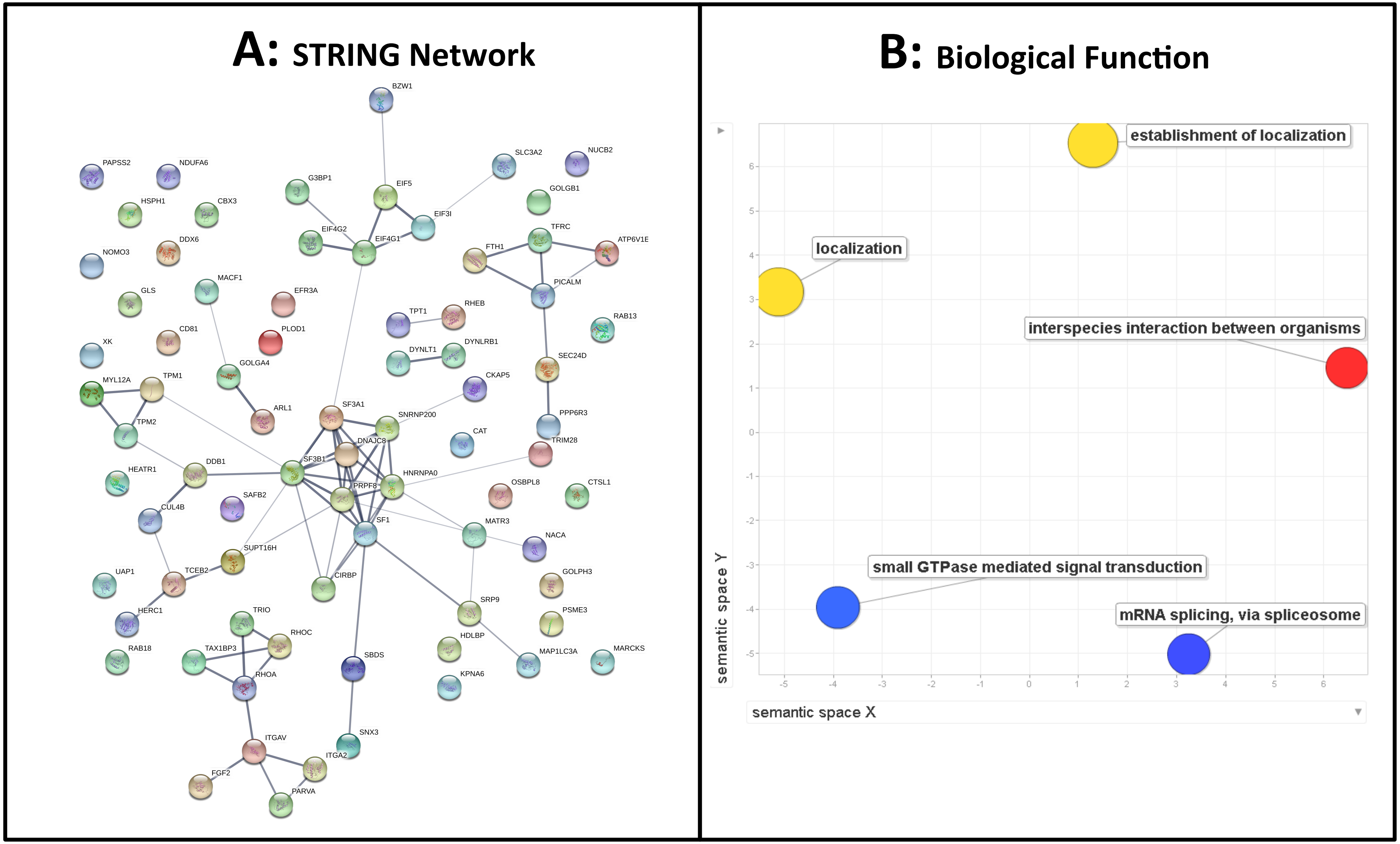
A: STRING Network
BZW1
PAPSS2
NDUFA6
SLC3A2
NUCB2
G3BP1
EIF5
HSPH1
CBX3
GOLGB1
EIF3I
DDX6
EIF4G2
EIF4G1
TFRC
NOMO3
FTH1
ATP6V1E
MACF1
GLS
EFR3A
PICALM
RHEB
TPT1
RAB13
CD81
PLOD1
XK
DYNLRB1
DYNLT1
GOLGA4
SEC24D
MYL12A
TPM1
CKAP5
ARL1
SF3A1
SNRNP200
PPP6R3
TPM2
CAT
DNAJC8
TRIM28
SF3B1
DDB1
HEATR1
HNRNPA0
PRPF8
OSBPL8
CTSL1
SAFB2
CUL4B
SF1
MATR3
NACA
SUPT16H
UAP1
TCEB2
CIRBP
GOLPH3
HERC1
TRIO
SRP9
PSME3
RHOC
HDLBP
RAB18
TAX1BP3
SBDS
MAP1LC3A
MARCKS
RHOA
KPNA6
ITGAV
SNX3
ITGA2
FGF2
PARVA
B: Biological Function
establishment of localization
localization
interspecies interaction between organisms
small GTPase mediated signal transduction
mRNA splicing, via spliceosome
semantic space Y
semantic space X
6
5
4
3
2
1
0
-1
-2
-3
-4
-5
-5
-4
-3
-2
-1
0
1
2
3
4
5
6

$30\ h \leq T_{1/2} < 40\ h$

A: STRING Network

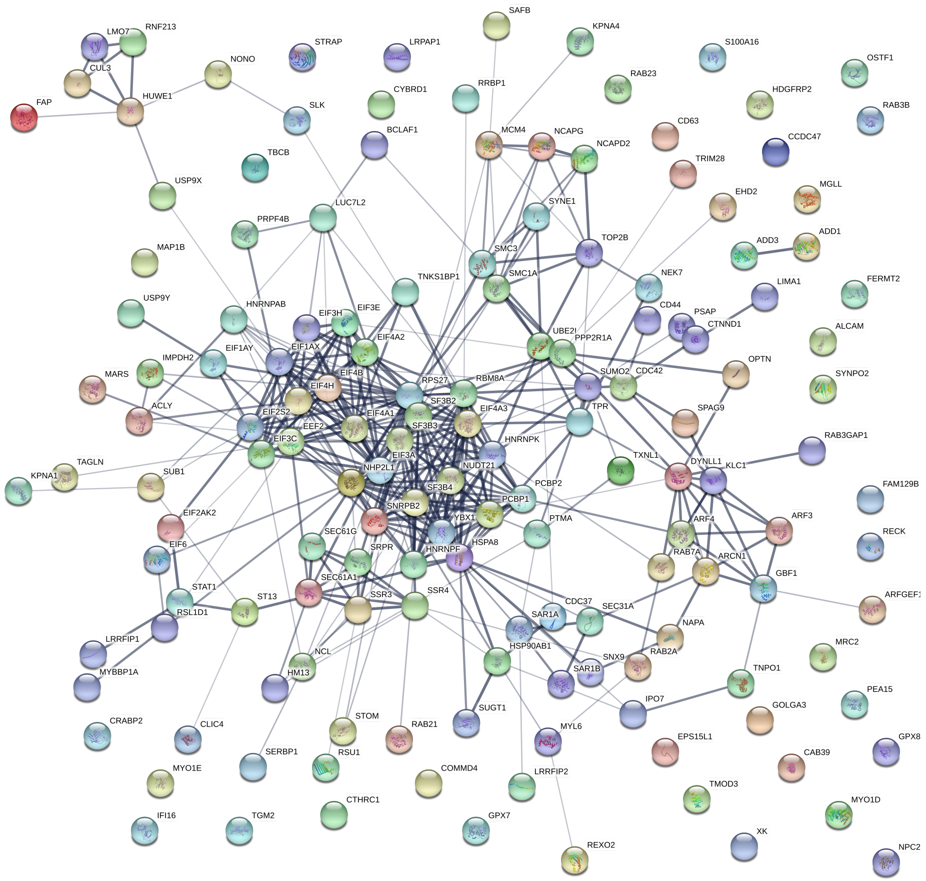

B: Biological Function

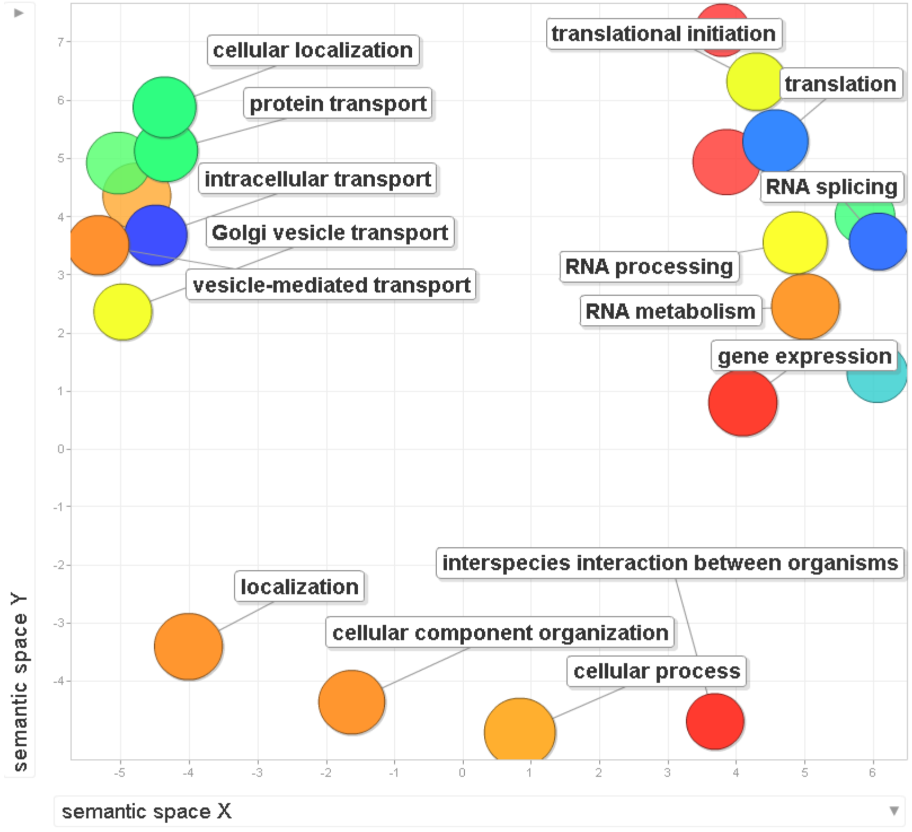

40 h ≤ $T_{1/2}$ < 50 h

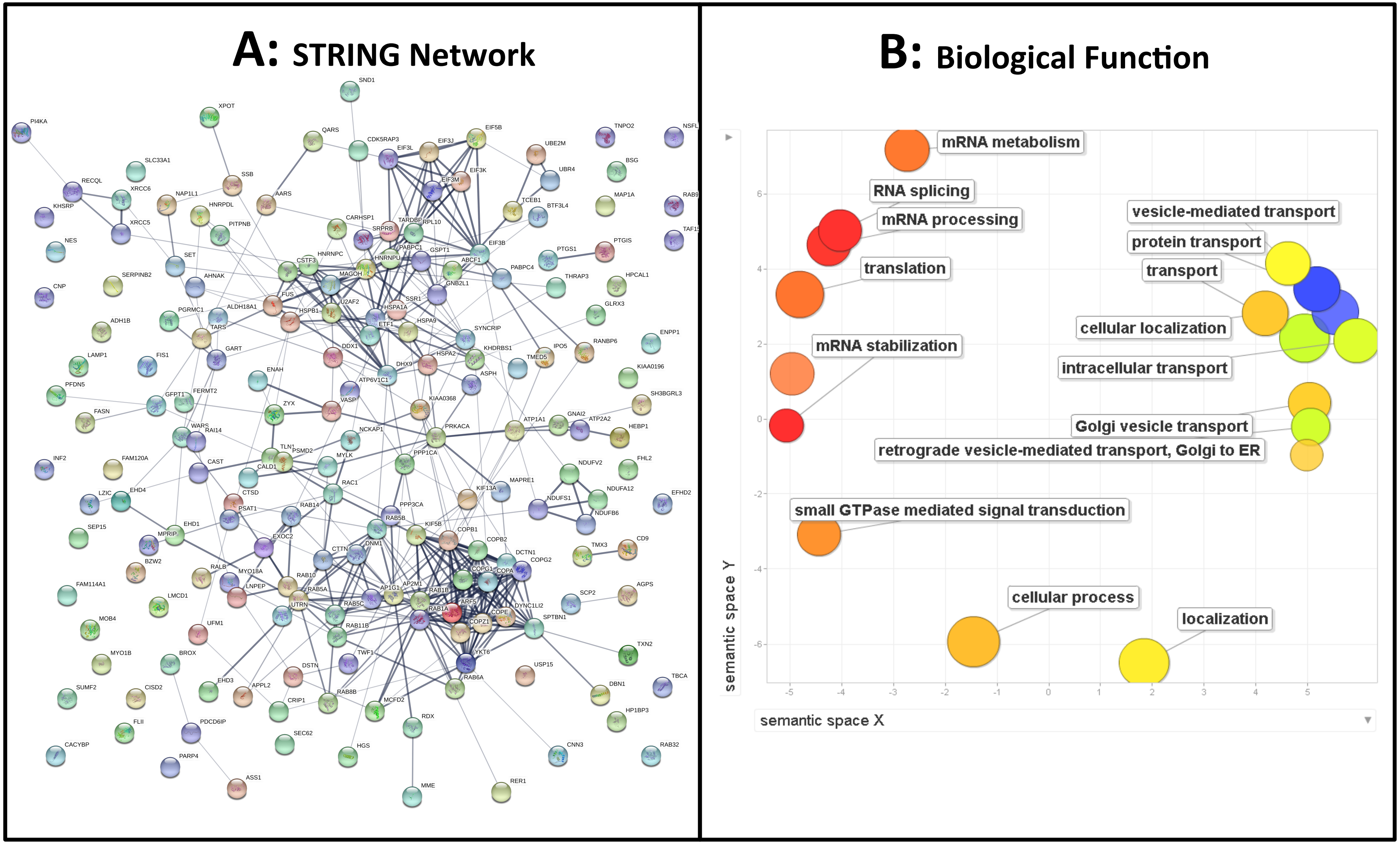
A: STRING Network
B: Biological Function
mRNA metabolism
RNA splicing
mRNA processing
translation
mRNA stabilization
vesicle-mediated transport
protein transport
transport
cellular localization
intracellular transport
Golgi vesicle transport
retrograde vesicle-mediated transport, Golgi to ER
small GTPase mediated signal transduction
cellular process
localization
semantic space Y
semantic space X
6
4
2
0
-2
-4
-6
-5
-4
-3
-2
-1
0
1
2
3
4
5

50 h ≤ $T_{1/2}$ < 60 h

A: STRING Network

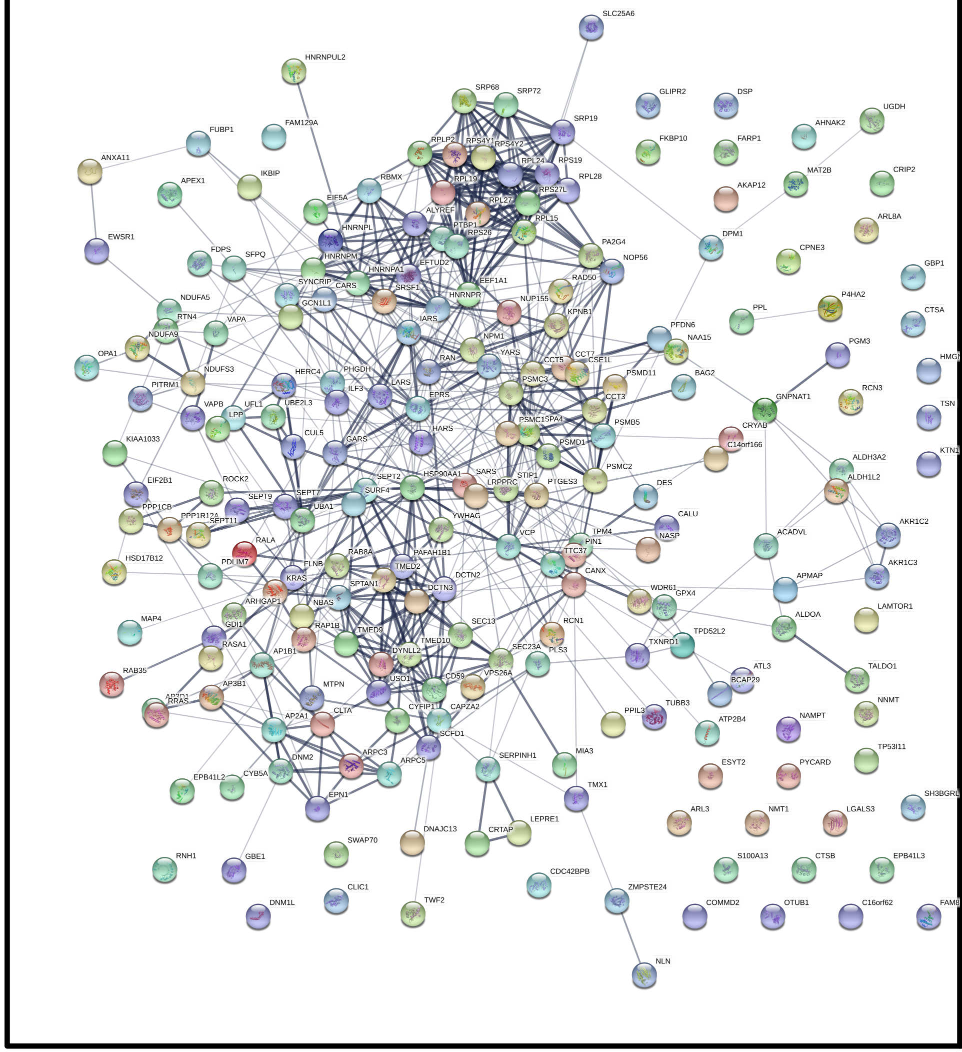

B: Biological Function

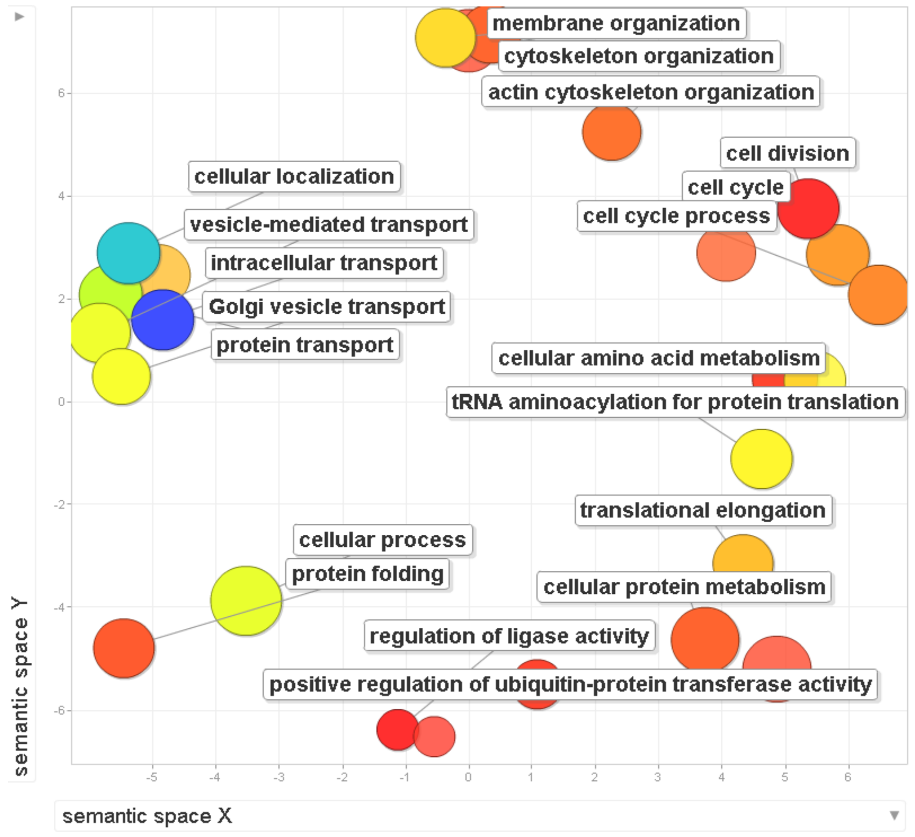

$60\ h \leq T_{1/2} < 70\ h$

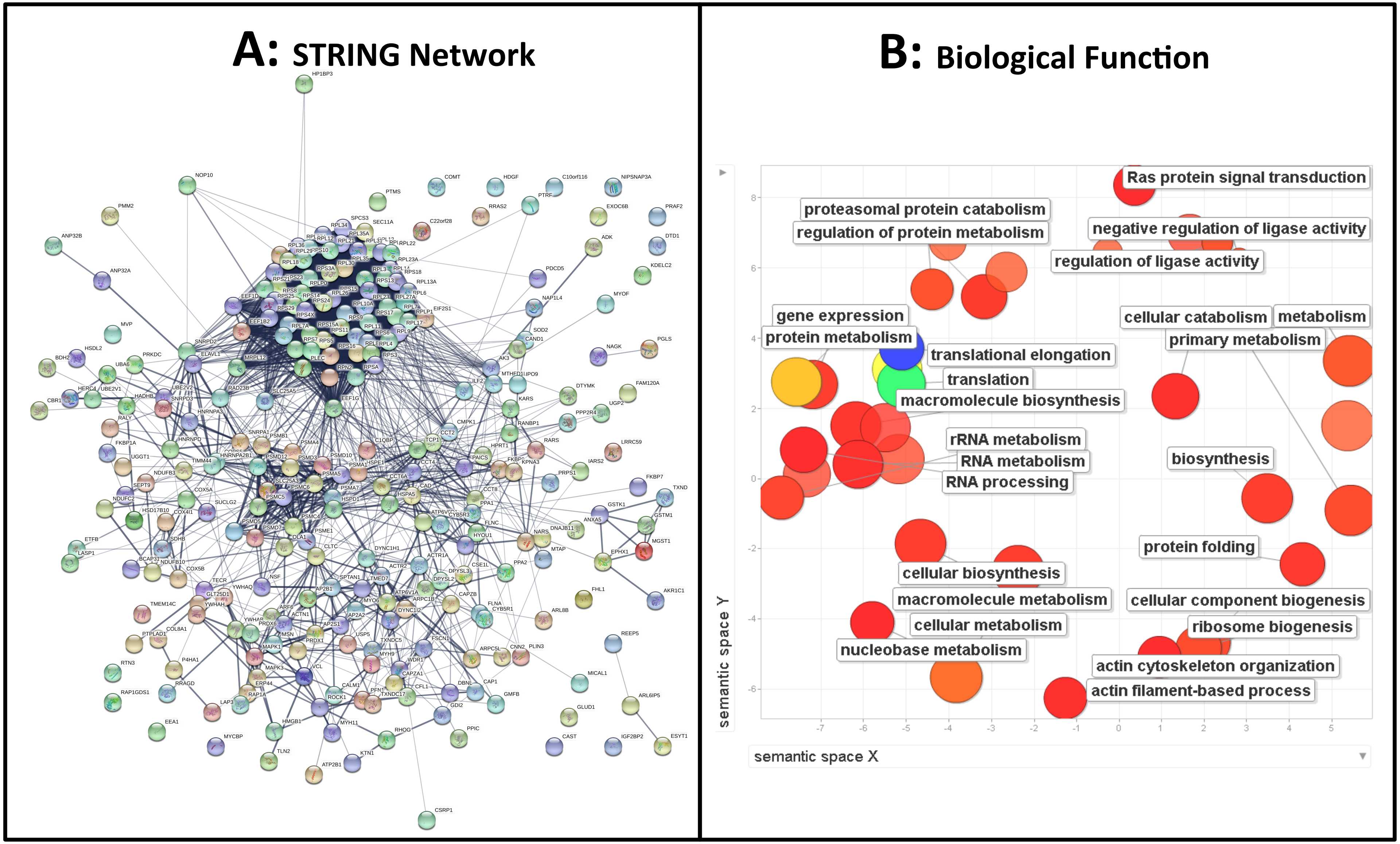

$70\ h \leq T_{1/2} < 80\ h$

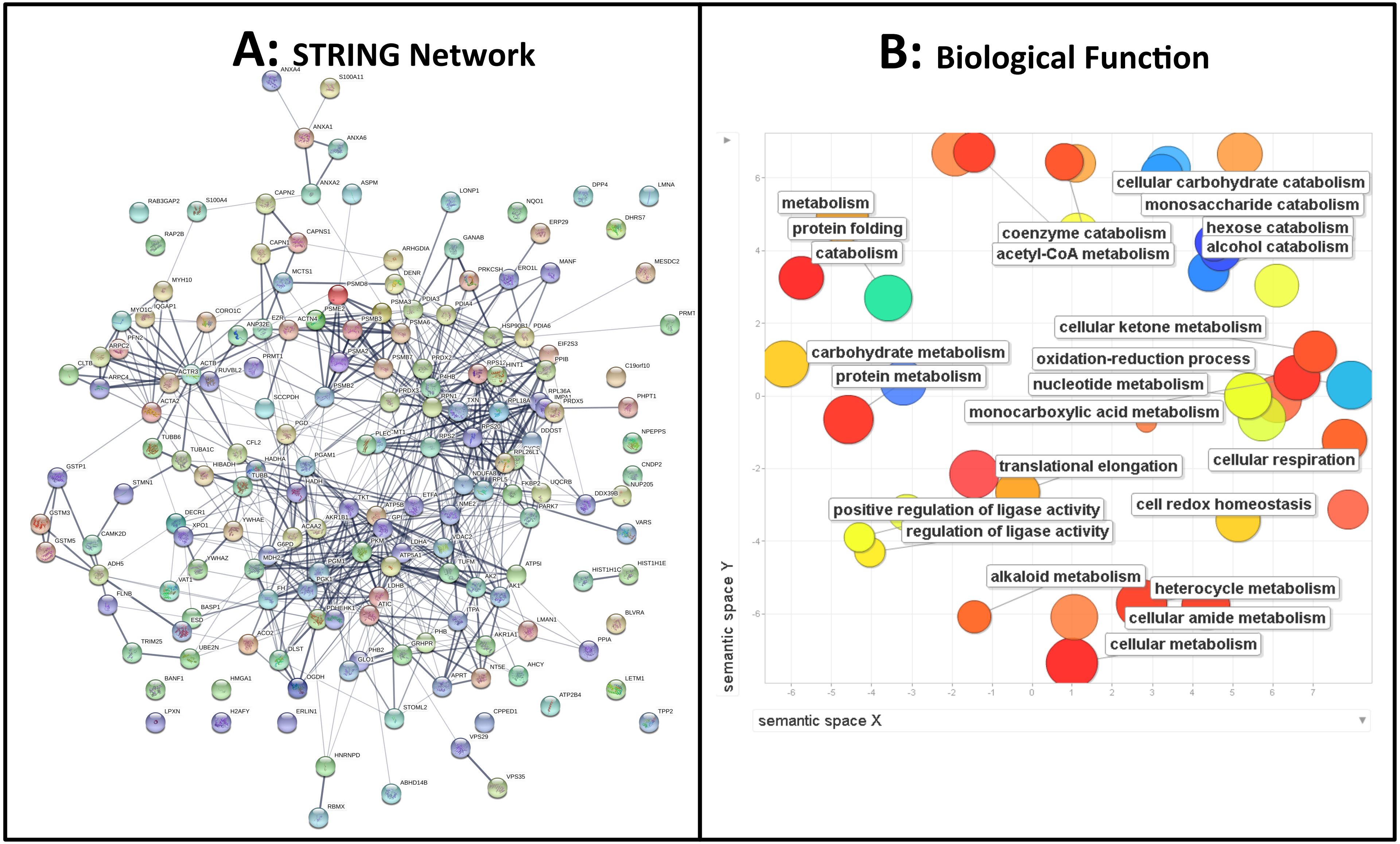

80 h ≤ $T_{1/2}$ < 90 h

A: STRING Network

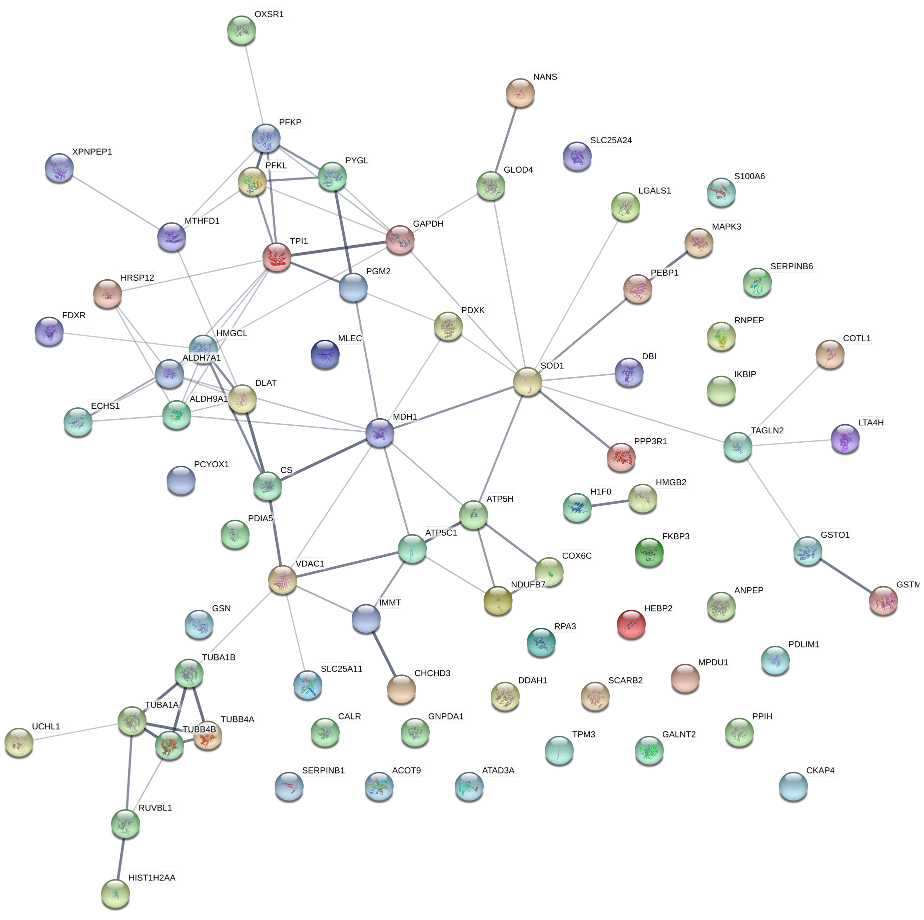

B: Biological Function

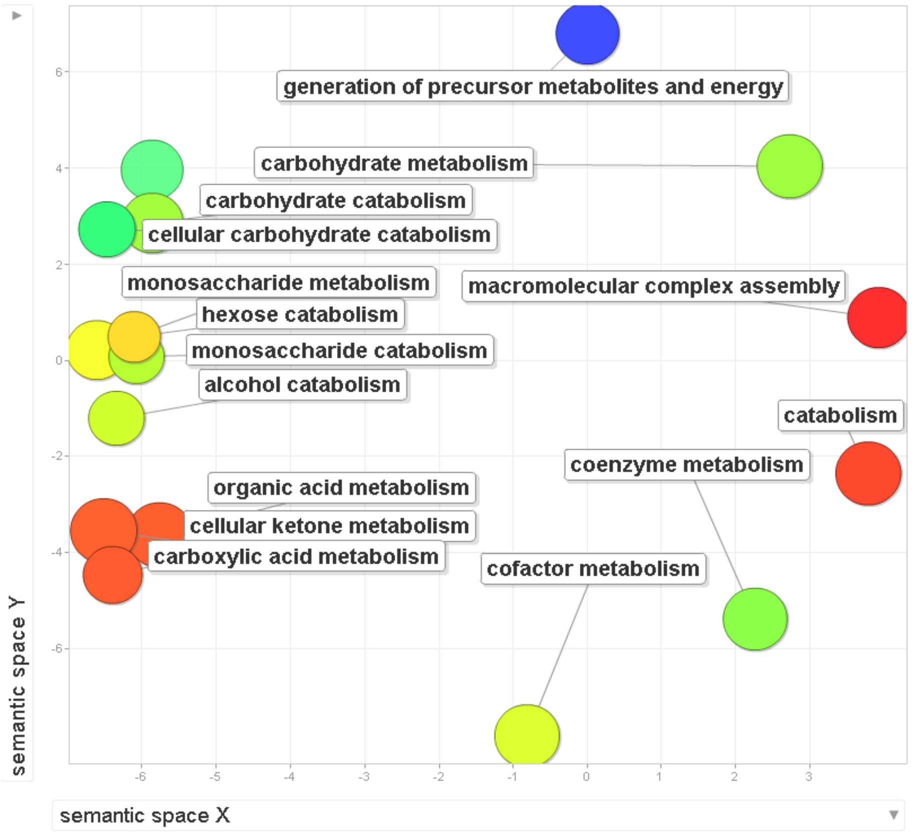

$T_{1/2} \geq 90$ h

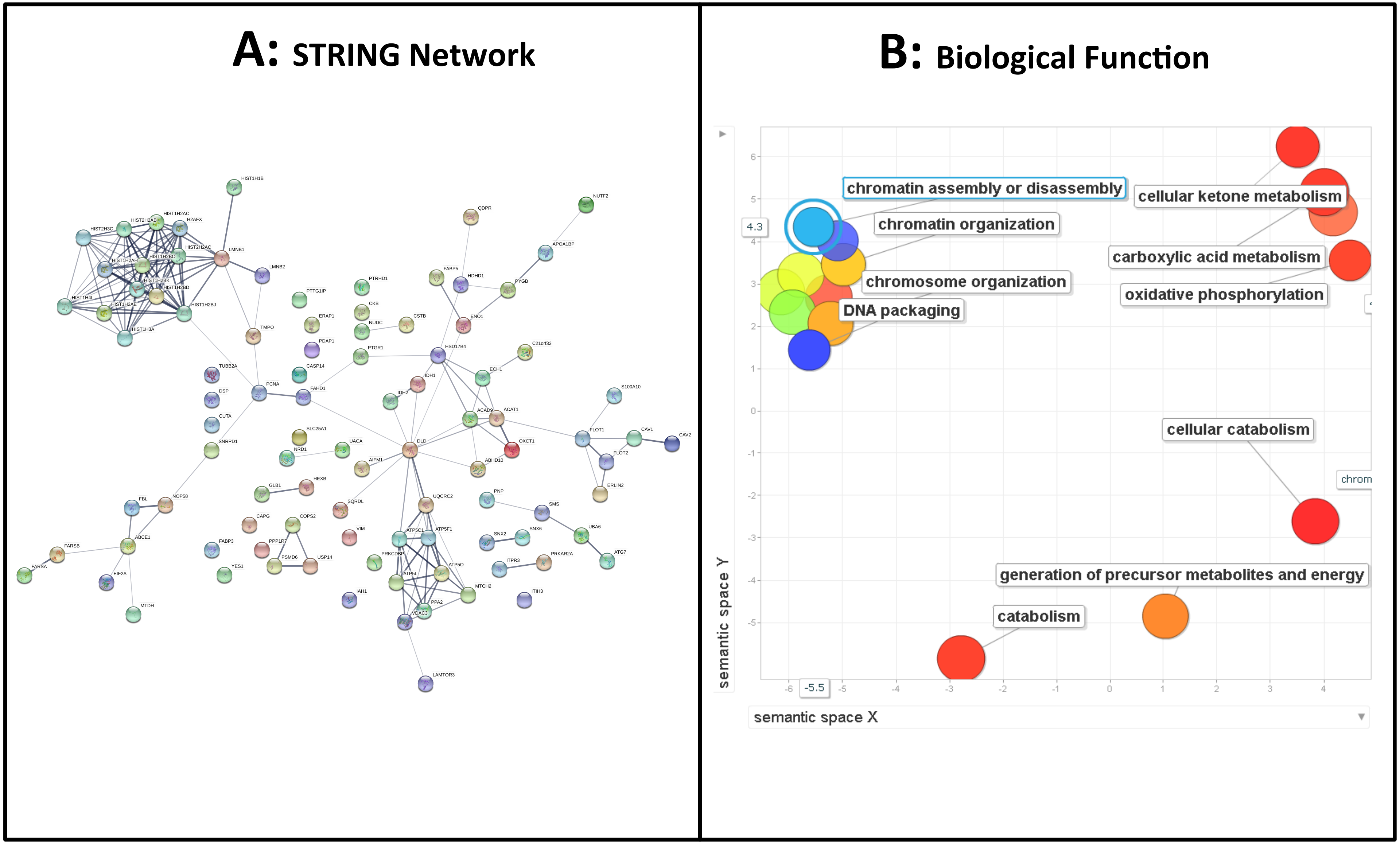
A: STRING Network
B: Biological Function
HIST1H1B
HIST1H2AC
HIST2H2AB
H2AFX
HIST2H3C
HIST2H2AC
LMNB1
HIST1H2AH
HIST1H2BO
LMNB2
HIST1H2BK
HIST1H2BD
HIST1H4I
HIST1H2AE
HIST1H2BJ
HIST1H3A
TMPO
PTTG1IP
ERAP1
PDAP1
CASP14
TUBB2A
DSP
CUTA
PCNA
FAHD1
SNRPD1
SLC25A1
NRD1
UACA
AIFM1
GLB1
HEXB
SQRDL
FBL
NOP58
ABCE1
FARSB
FARSA
EIF2A
MTDH
CAPG
COPS2
FABP3
PPP1R7
PSMD6
USP14
YES1
VIM
IAH1
PTRHD1
CKB
NUDC
CSTB
PTGR1
QDPR
FABP5
HDHD1
PYGB
APOA1BP
NUTF2
ENO1
HSD17B4
C21orf33
ECH1
IDH1
IDH2
ACAD9
ACAT1
OXCT1
DLD
ABHD10
S100A10
FLOT1
CAV1
CAV2
FLOT2
ERLIN2
UQCRC2
PNP
SMS
ATP5C1
ATP5F1
SNX2
SNX6
UBA6
PRKCDBP
ATP5O
ITPR3
PRKAR2A
ATG7
ATP5L
MTCH2
PPA2
VDAC3
ITIH3
LAMTOR3
chromatin assembly or disassembly
cellular ketone metabolism
chromatin organization
carboxylic acid metabolism
chromosome organization
oxidative phosphorylation
DNA packaging
cellular catabolism
chrom
generation of precursor metabolites and energy
catabolism
4.3
-5.5
semantic space Y
semantic space X
6
5
4
3
2
1
0
-1
-2
-3
-4
-5
-6
-4
-3
-2
-1
0
1
2
3
4
