# Supplementary material for: Modeling SILAC Data to Assess Protein Turnover in a Cellular Model of Diabetic Nephropathy
Source: Int J Mol Sci. 2023 Feb 1;24(3):2811. doi: 10.3390/ijms24032811 (PMC9917874; doi:10.3390/ijms24032811)

Log2 Conc ≥ 9.0 (arbitrary units)

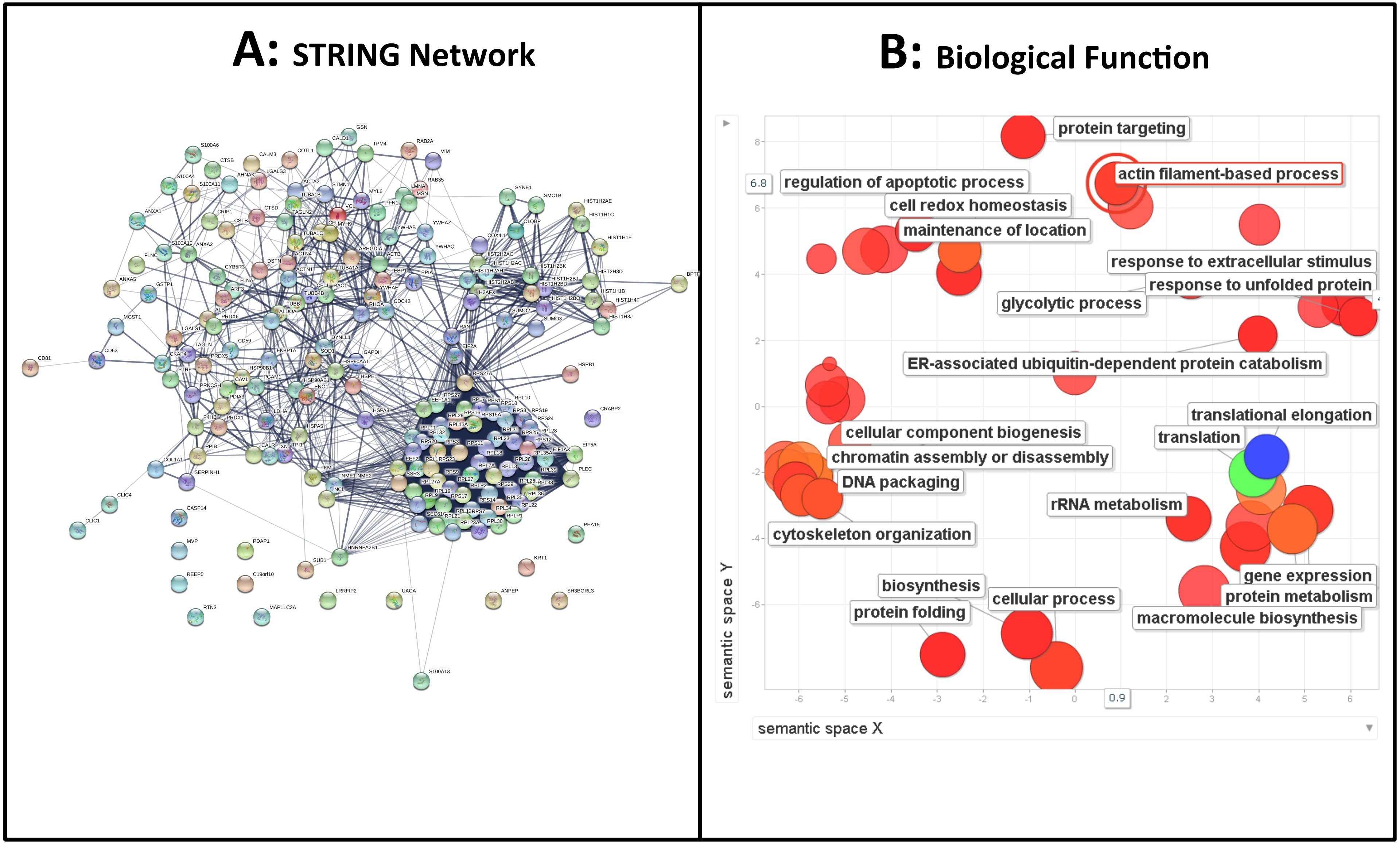

8.0 ≤ Log2 Conc < 9.0 (arbitrary units)

A: STRING Network

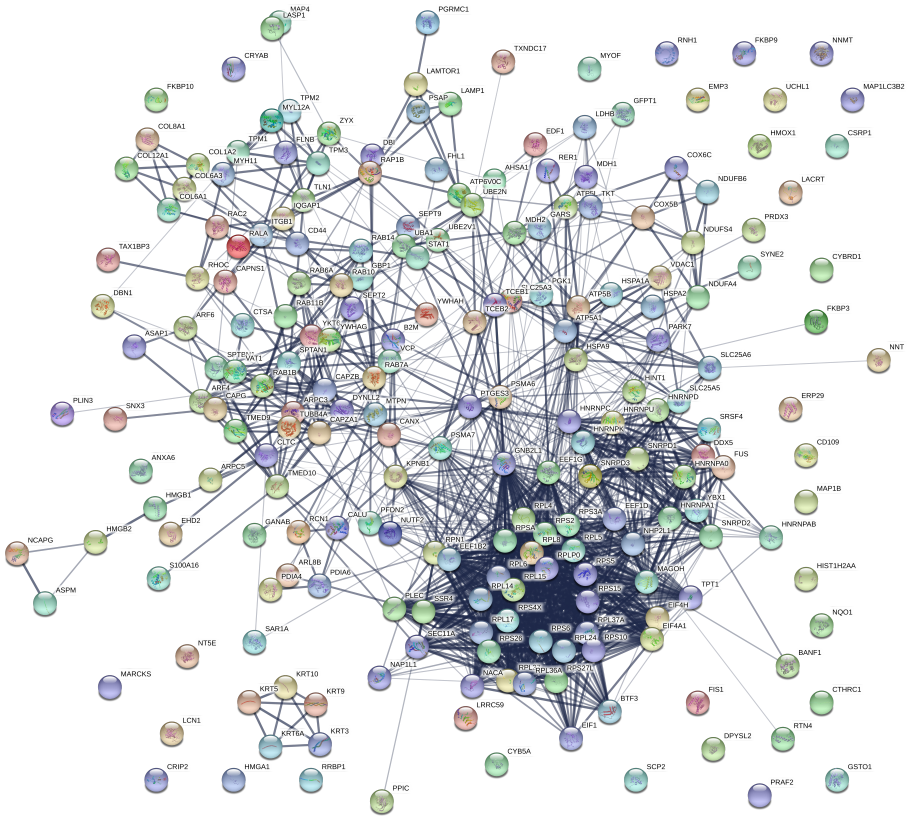

B: Biological Function

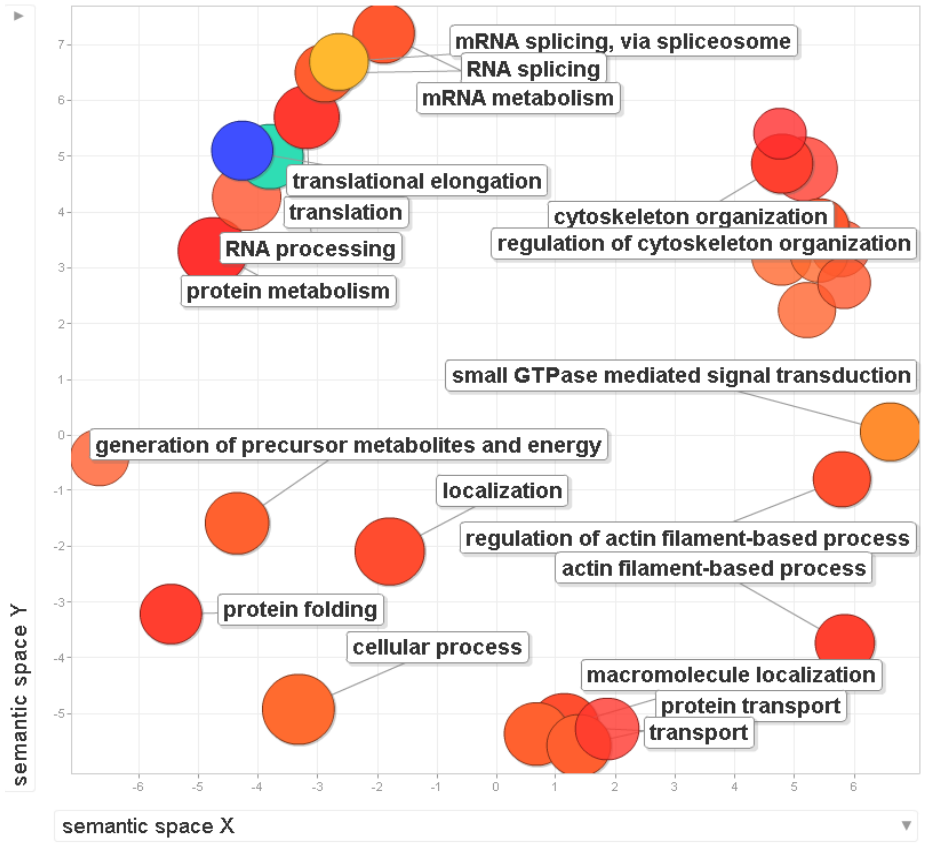

7.5 ≤ Log2 Conc < 8.0 (arbitrary units)

A: STRING Network

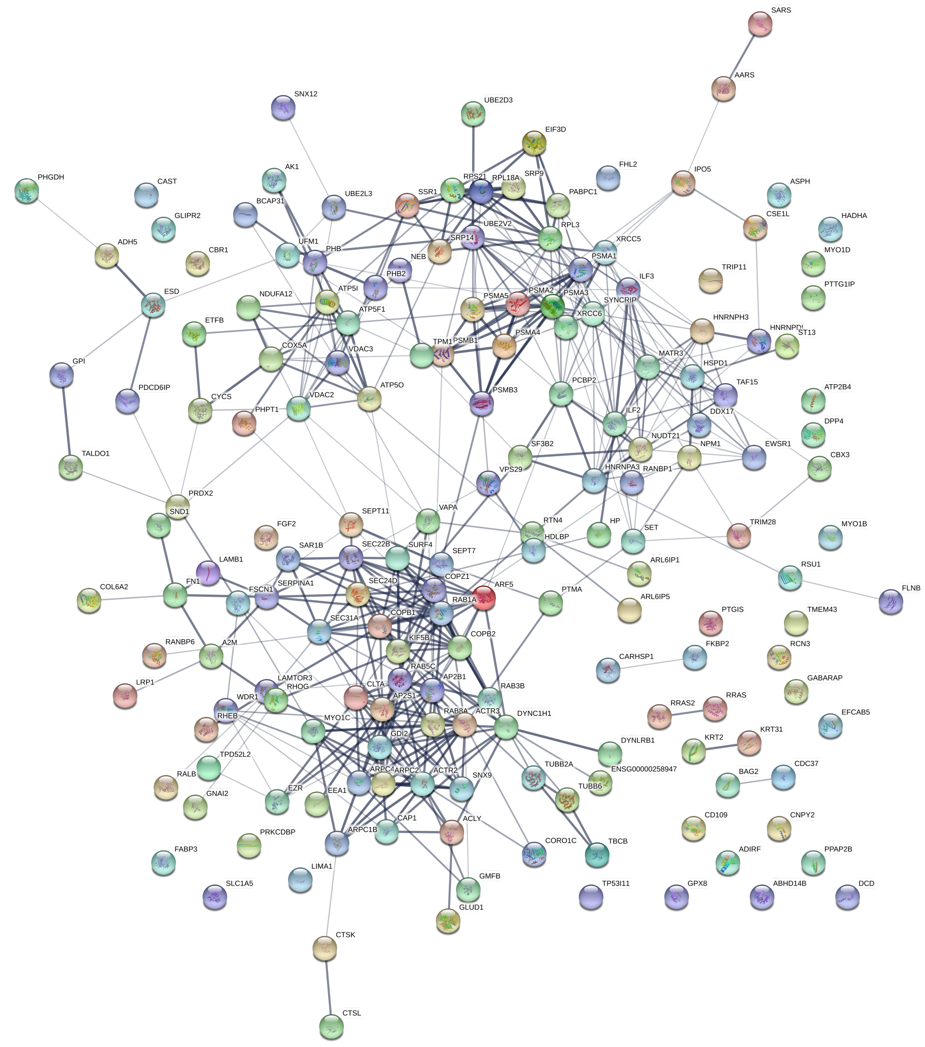

B: Biological Function

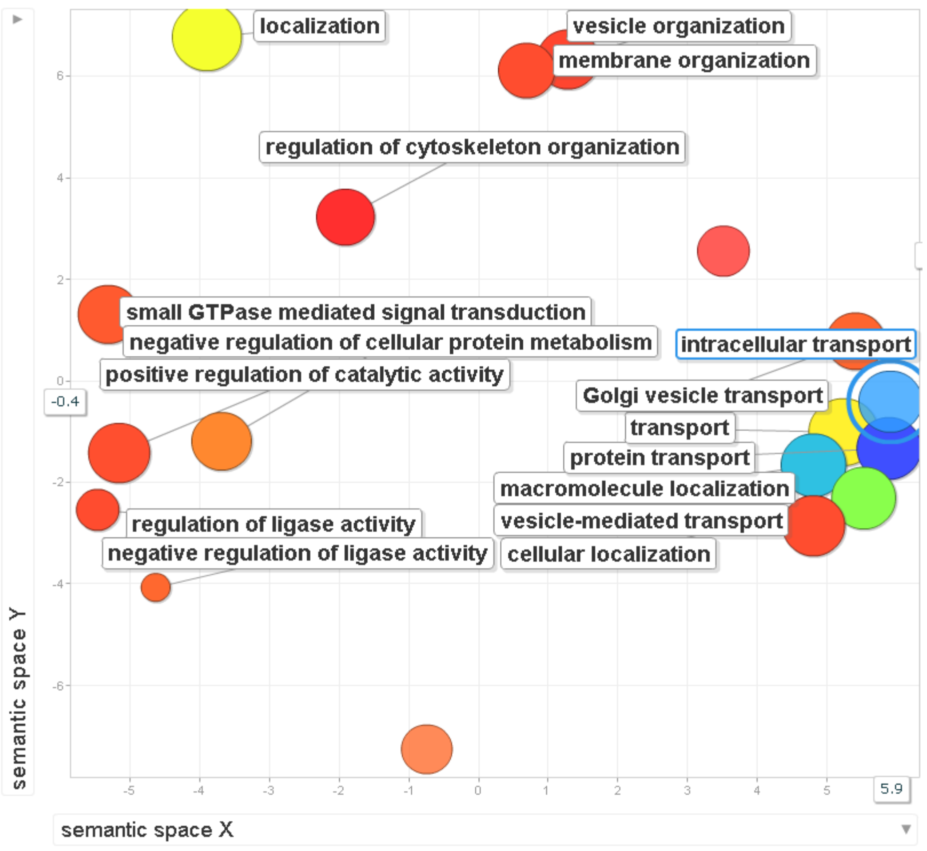

7.0 ≤ Log2 Conc < 7.5 (arbitrary units)

A: STRING Network

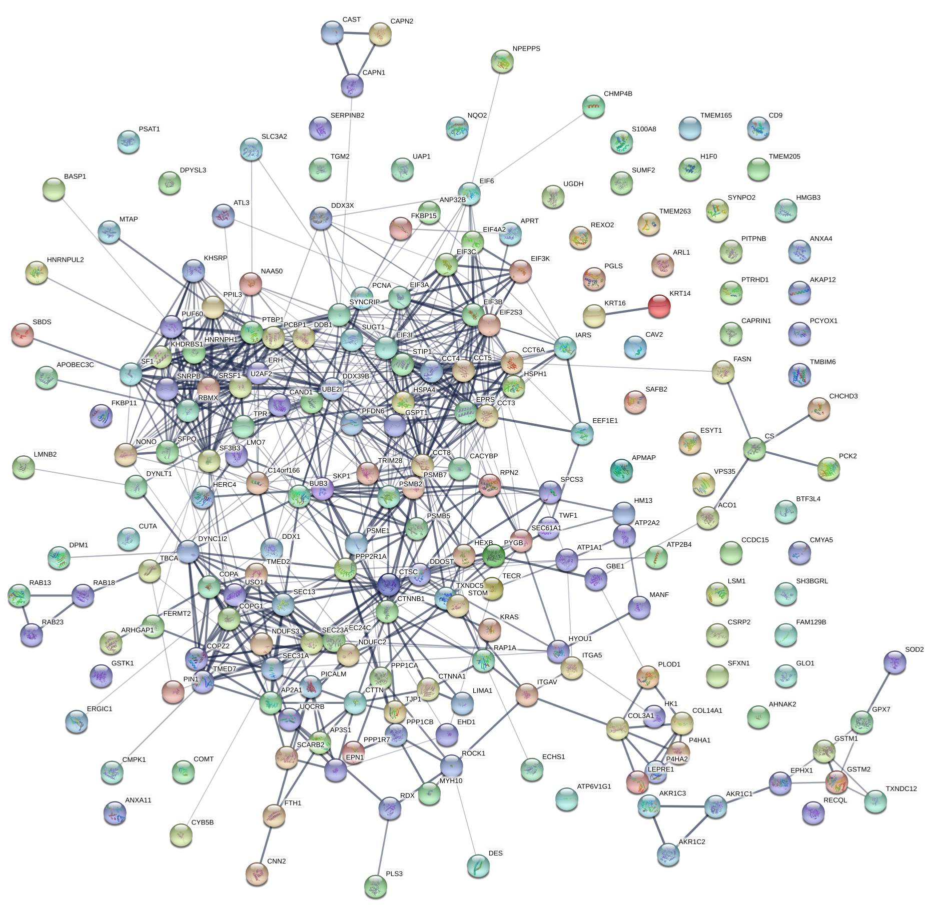

B: Biological Function

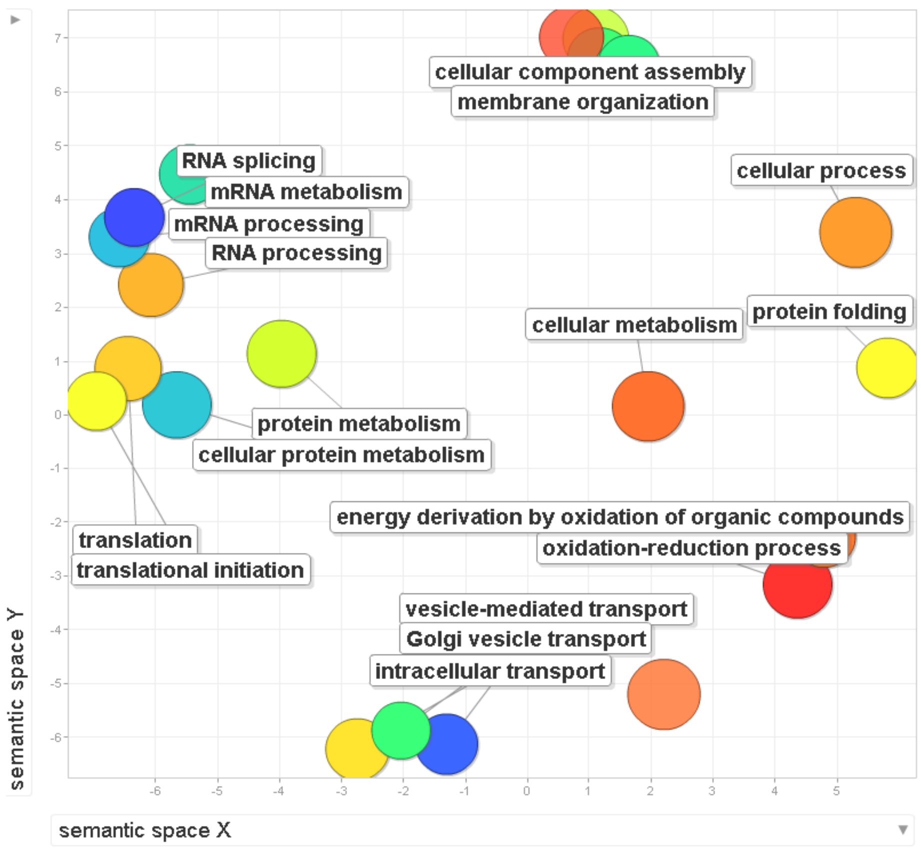

6.5 ≤ Log2 Conc < 7.0 (arbitrary units)

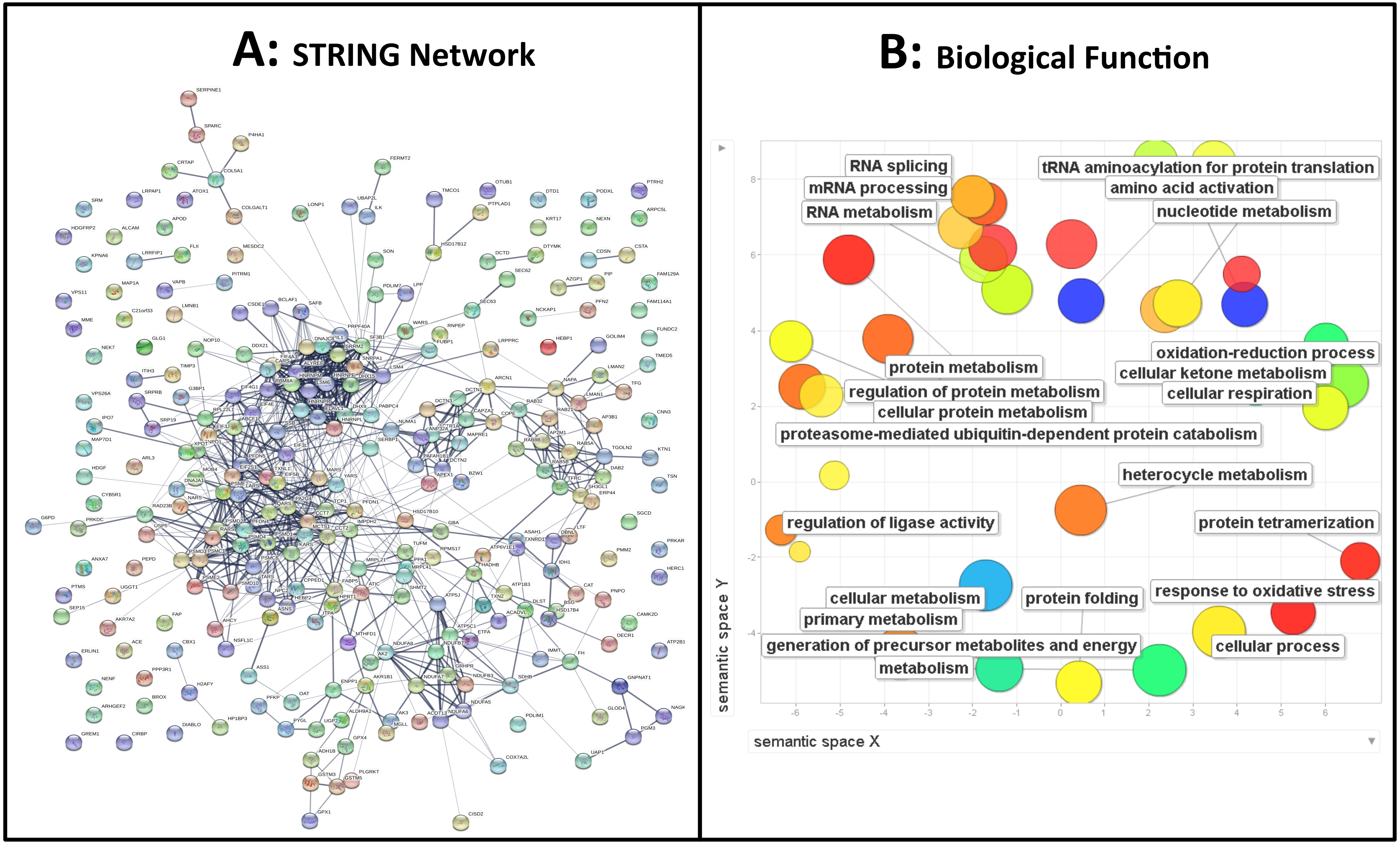

6.0 ≤ Log2 Conc < 6.5 (arbitrary units)

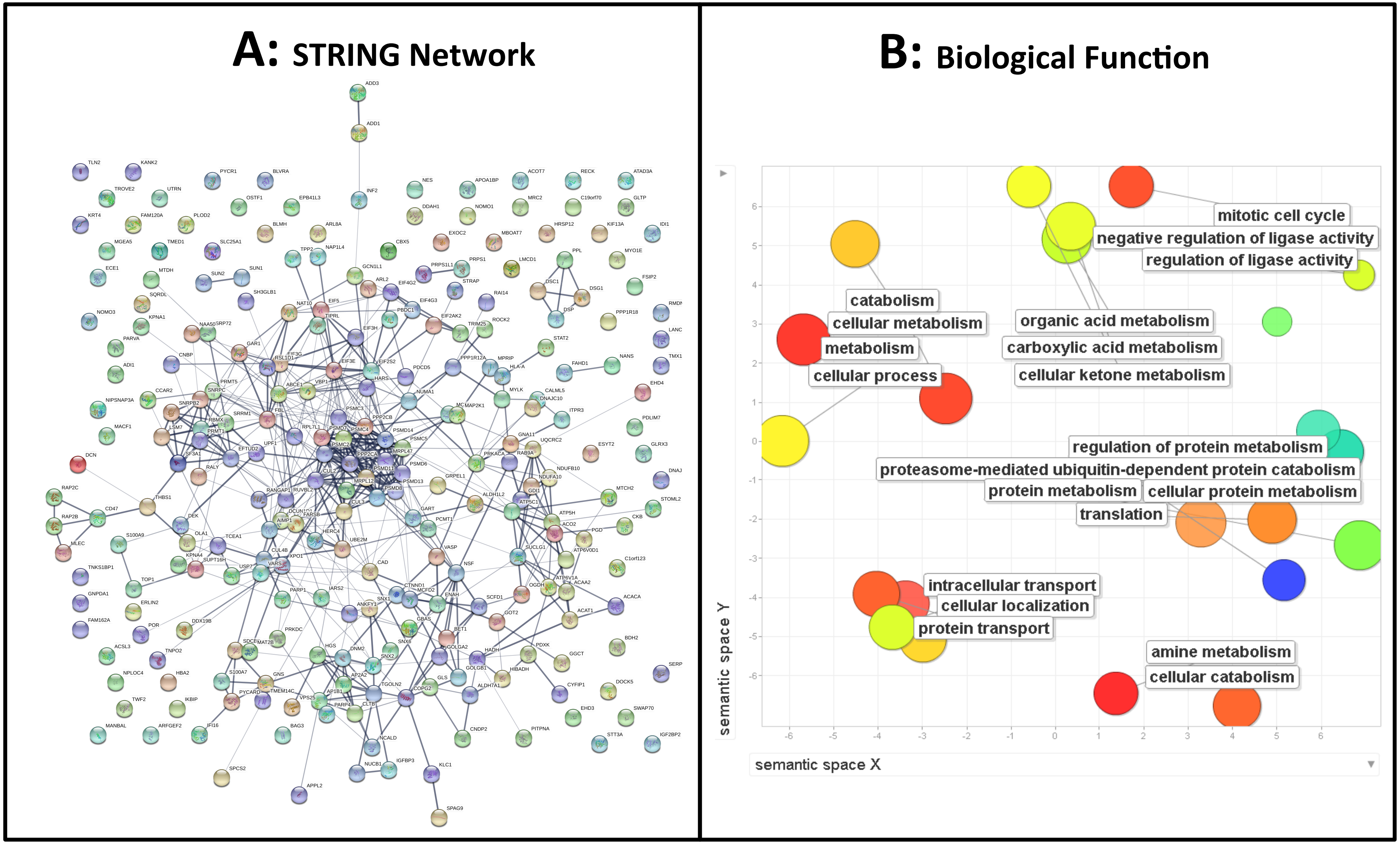

5.0 ≤ Log2 Conc < 6.0 (arbitrary units)

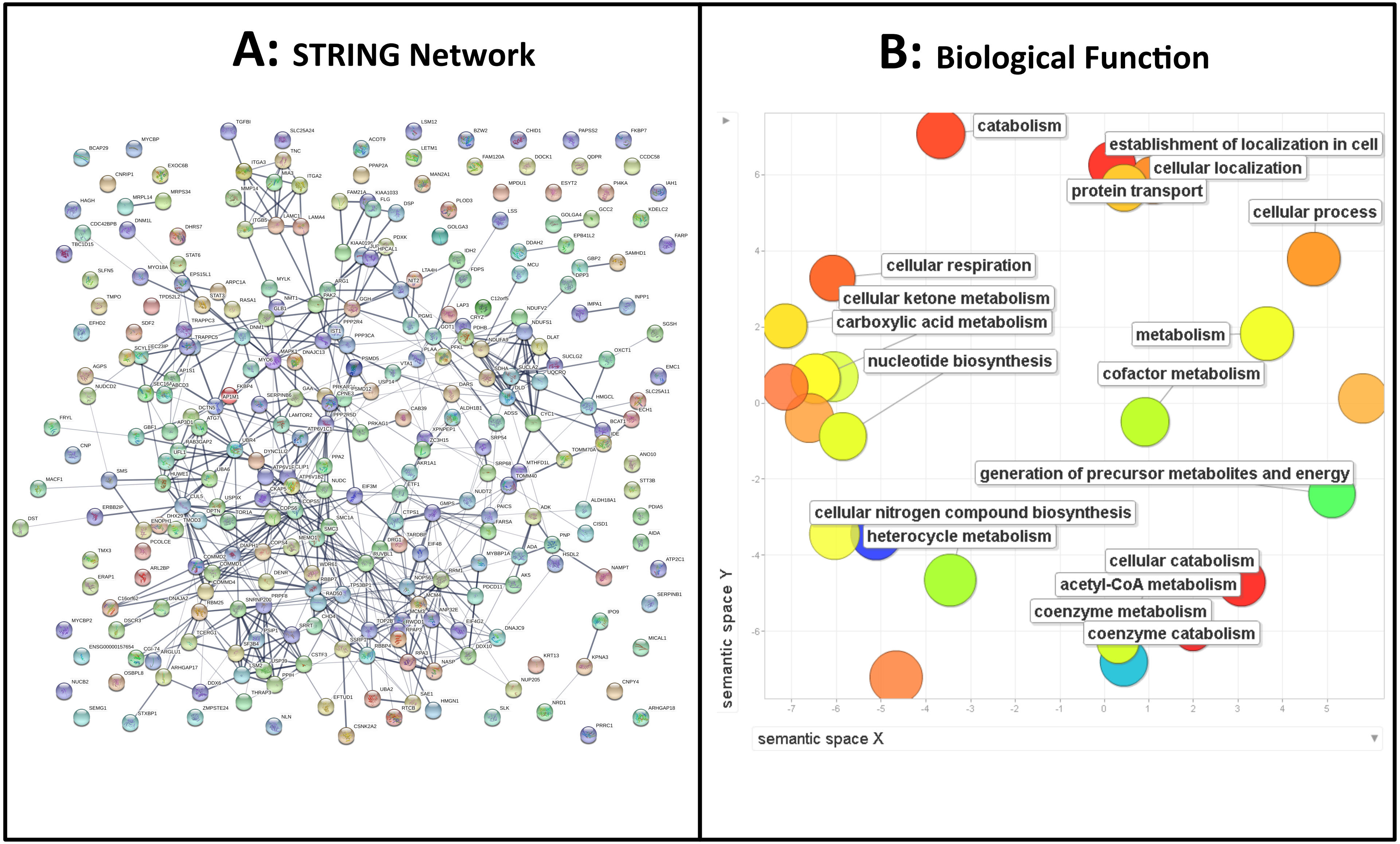

Log2 Conc < 5.0 (arbitrary units)

A: STRING Network

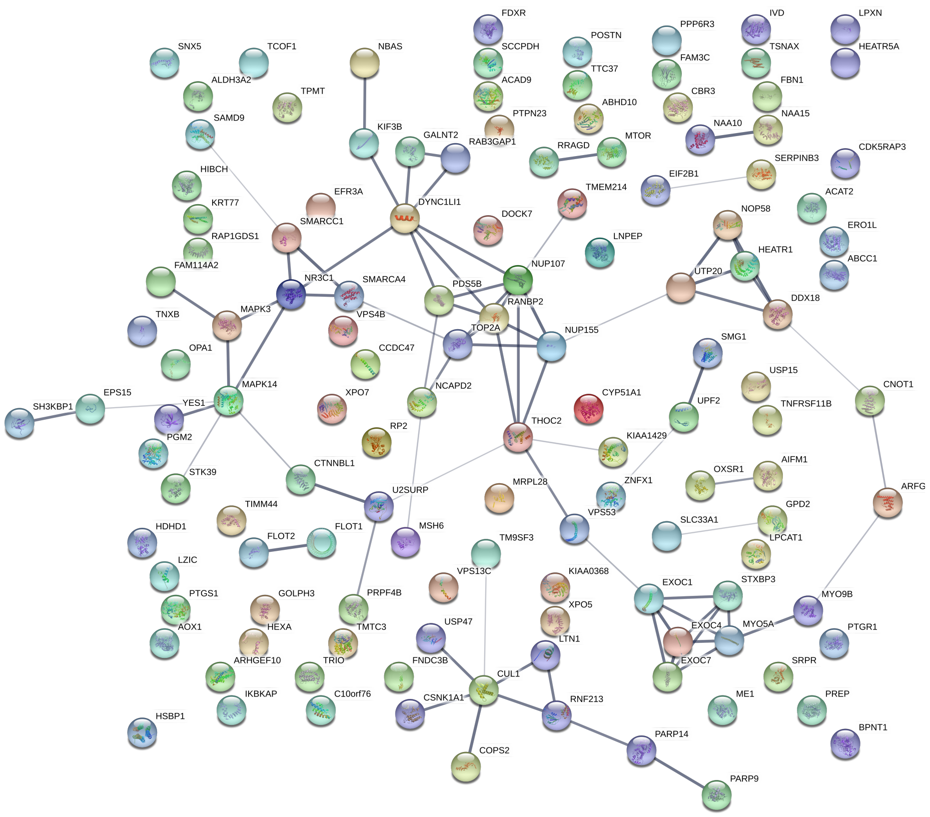

B: Biological Function

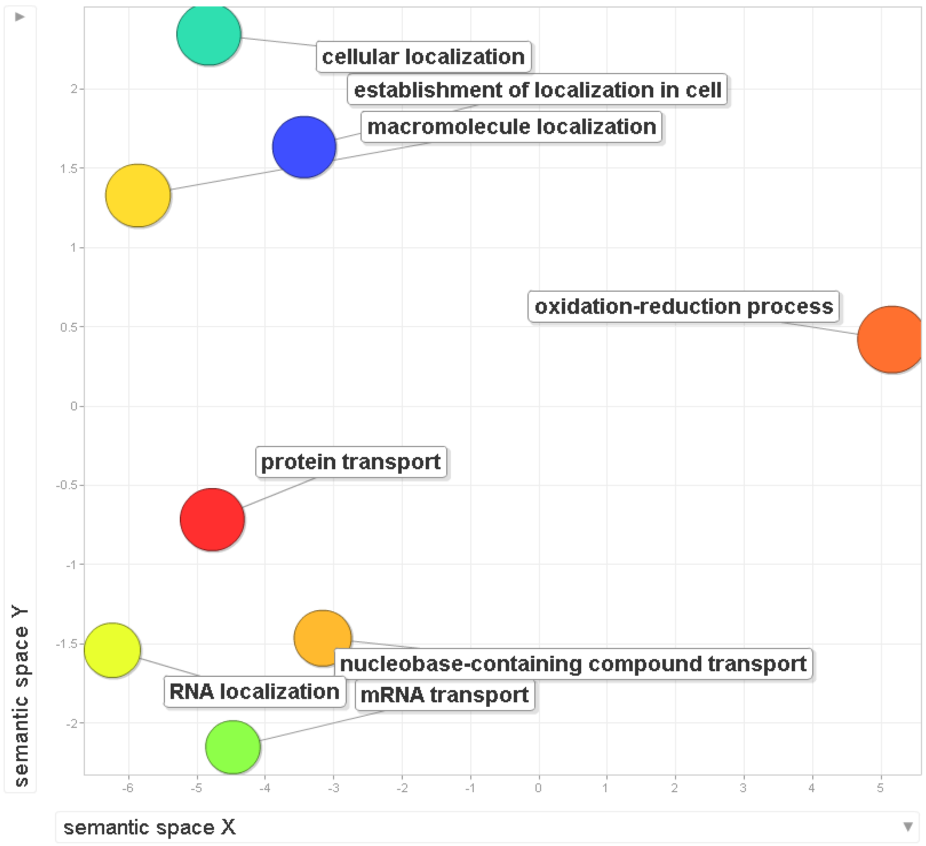

Supplement: Supplementary file 1 [file ijms-24-02811-s001.zip › Supplementary material/Figure S2.pdf]
