# Supplementary material for: Modeling SILAC Data to Assess Protein Turnover in a Cellular Model of Diabetic Nephropathy
Source: Int J Mol Sci. 2023 Feb 1;24(3):2811. doi: 10.3390/ijms24032811 (PMC9917874; doi:10.3390/ijms24032811)
Supplement: Supplementary file 1 [file ijms-24-02811-s001.zip › Supplementary material/Figure S3.pdf]

Group 1: proteins with low turnover rate k (long half-life) and high relative abundance

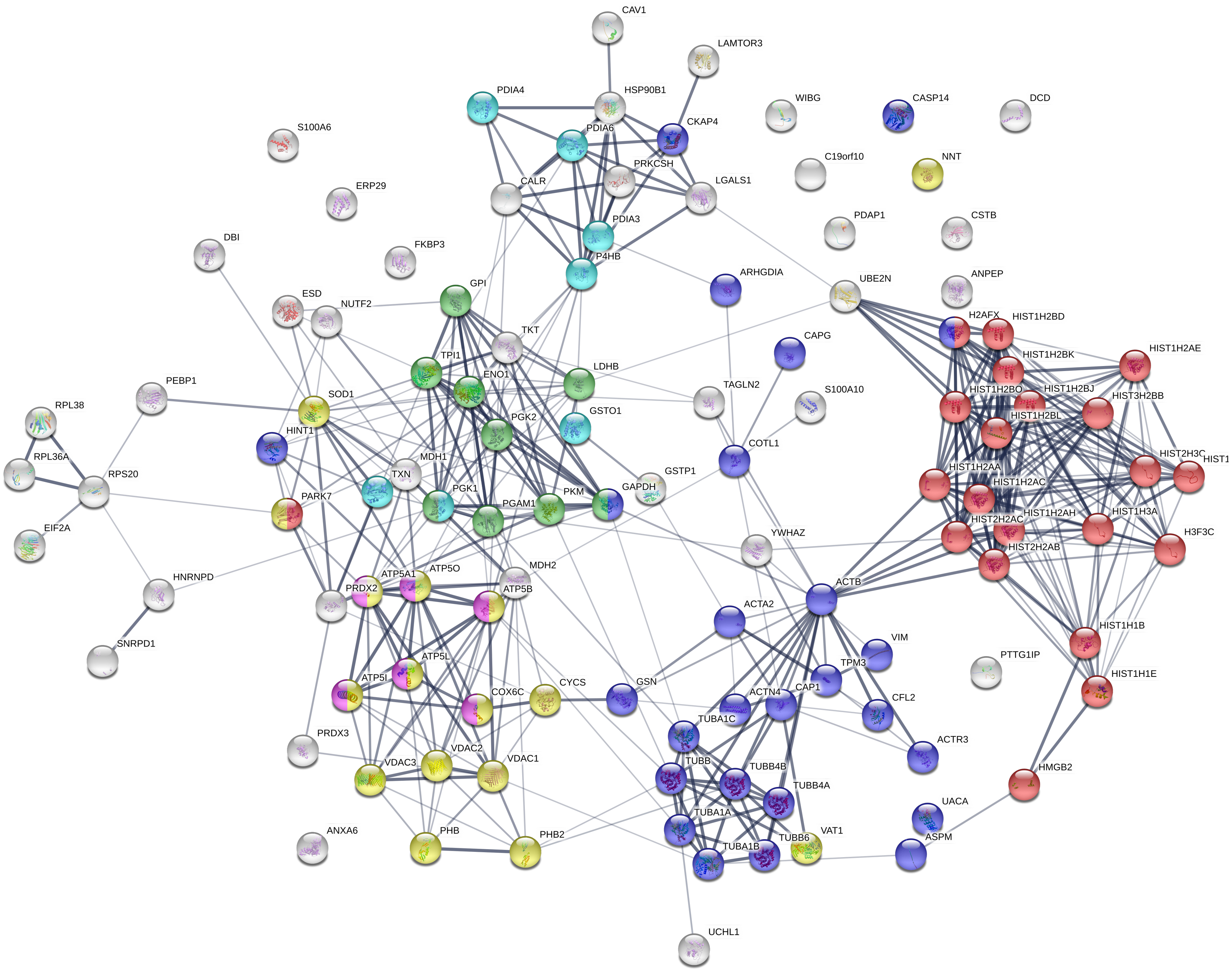

Group 1: proteins with low turnover rate k (long half-life) and high relative abundance

Cytoskeleton	(Cellular Component; FDR 7.0 e-4)
Chromatin	(Cellular Component; FDR 2.77 e-11)
Glycolysis/Gluconeogenesis	(KEGG pathways; FDR 1.44 e-8)
Mitochondrial envelope	(Cellular Component; FDR 4.09 e-5)
Disulfide oxidoreductase activity	(Molecular Function; FDR 7.38 e-7)
Oxidative phosphorylation	(KEGG pathways; FDR 1.1e-3)

Group 2: proteins with high turnover rate (short half-life) and high relative abundance

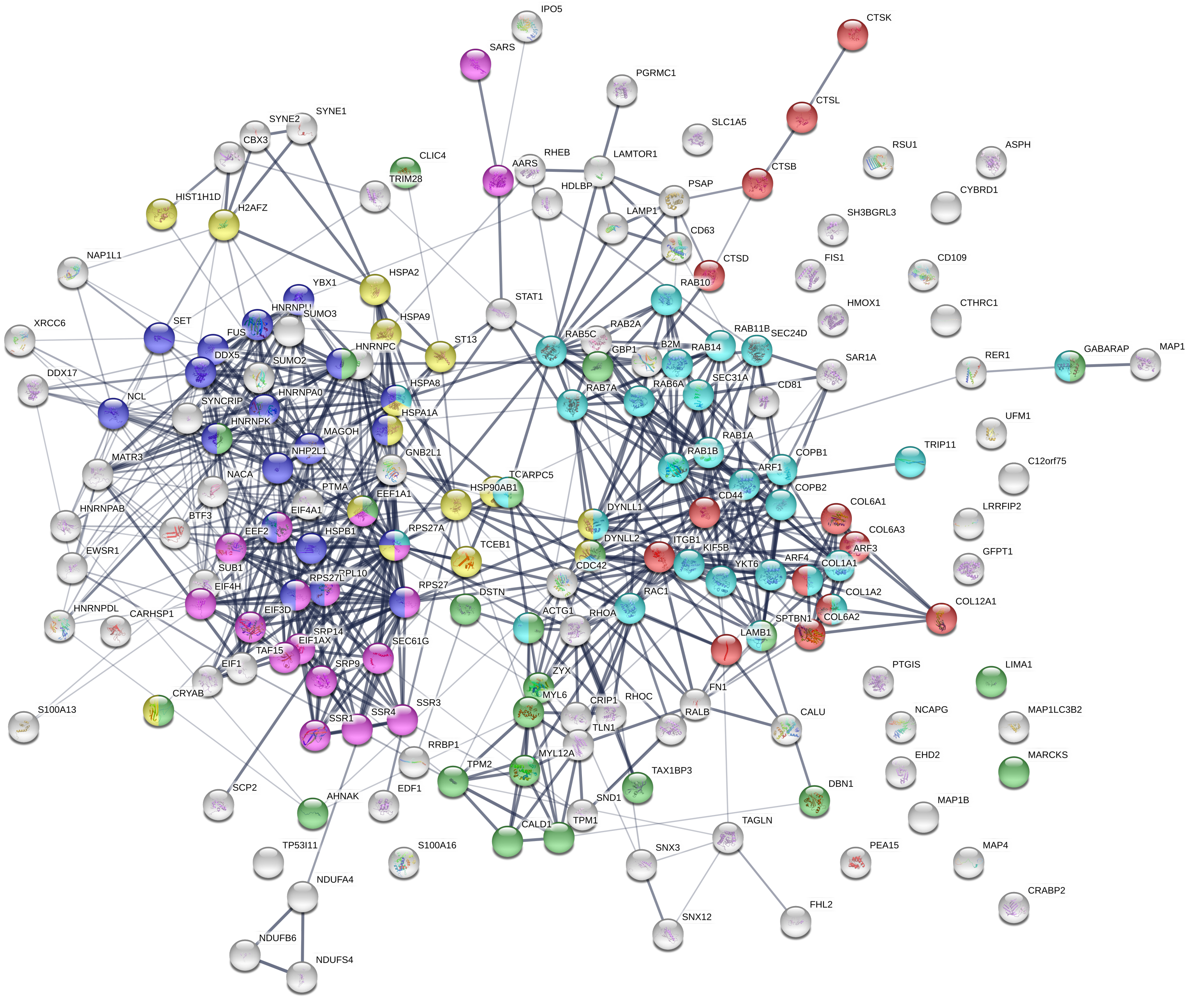

Group 2: proteins with high turnover rate (short half-life) and high relative abundance

**Metabolism of mRNA**	(Reactome; FDR 1.5 e-4)
**Extracellular matrix organization**	(Reactome; FDR 9.06 e-5)
**Actin cytoskeleton**	(Cellular Component; FDR 3.86 e-10)
**Cellular responses to stress**	(Reactome; FDR 6.53 e-5)
**Vesicle mediated transport**	(Reactome; FDR 4.18 e-10)
**Translation**	(Reactome; FDR 2.77 e-8)

Group 3: proteins with high turnover rate (short half-life) and low relative abundance

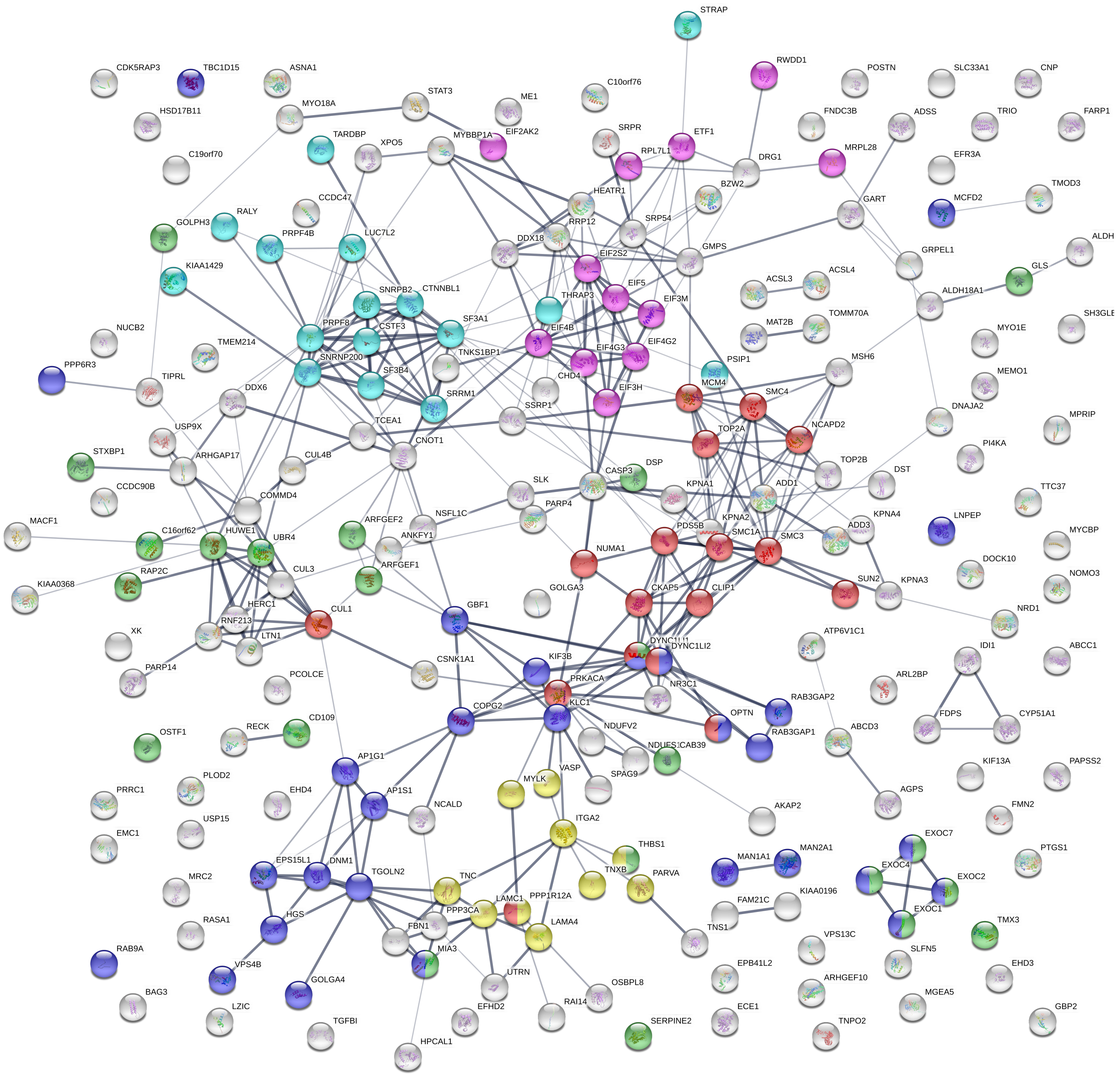

Group 3: proteins with high turnover rate (short half-life) and low relative abundance

**Membrane trafficking**	(Reactome; FDR 2.4 e-7)
**Cell Cycle**	(Reactome; FDR 2.0 e-2)
**Secretion by cell**	(Biological Process; FDR 4.8 e-2)
**Focal adhesion**	(KEGG; FDR 1.57 e-2)
**RNA splicing**	(Biological Process; FDR 1.4 e-3)
**Translation**	(Biological Process; FDR 3.4 e-2)

Group 4: proteins with low turnover rate k (long half-life) and low relative abundance

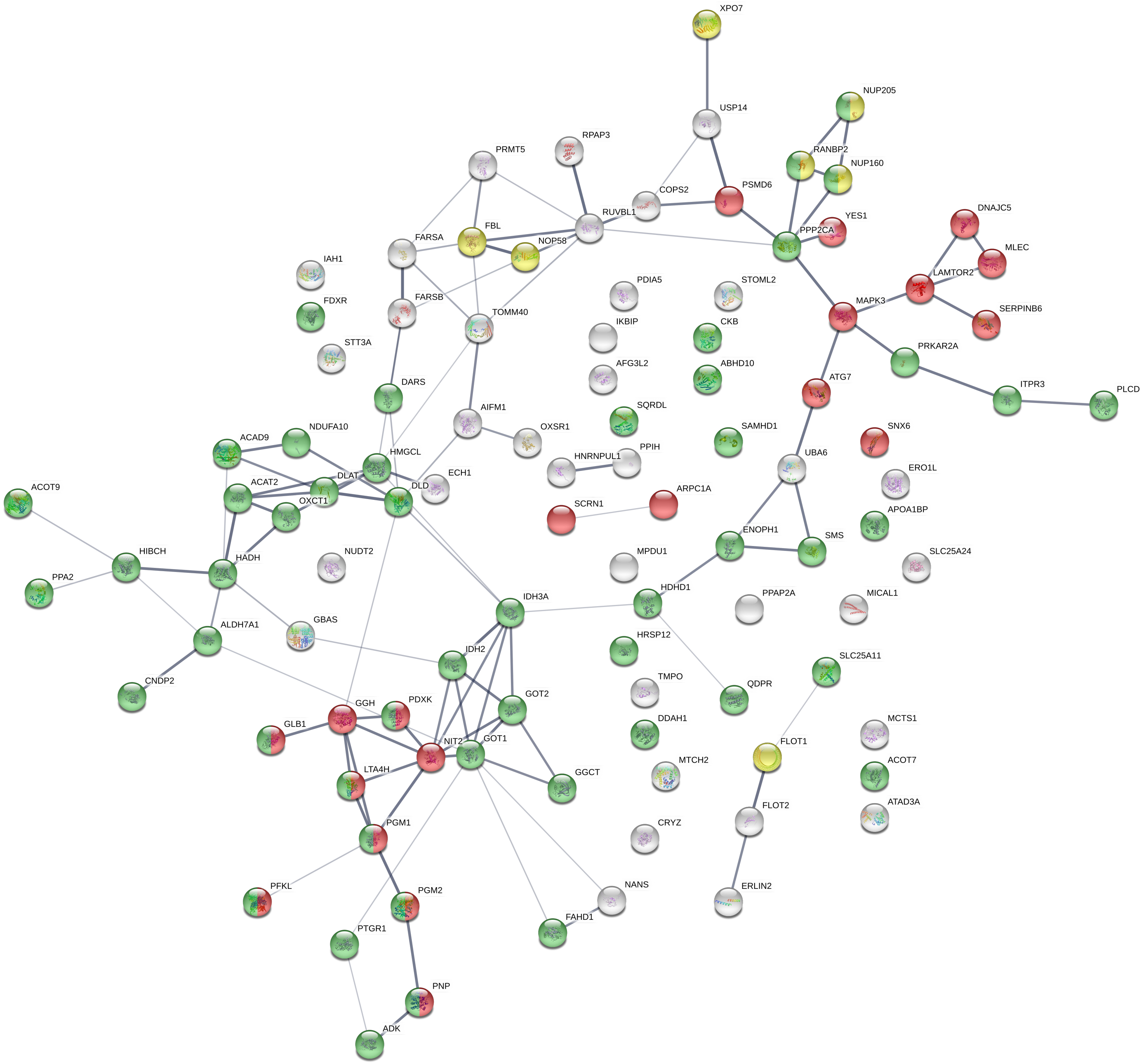

Group 4: proteins with low turnover rate k (long half-life) and low relative abundance

**Vesicle mediated transport**	(Biological Process; FDR 6.6 e-3)
**Metabolism**	(Reactome; FDR 6.91 e-20)
**RNA localization**	(Biological Process; FDR 2.7 e-3)
