# Supplementary material for: Modeling SILAC Data to Assess Protein Turnover in a Cellular Model of Diabetic Nephropathy
Source: Int J Mol Sci. 2023 Feb 1;24(3):2811. doi: 10.3390/ijms24032811 (PMC9917874; doi:10.3390/ijms24032811)
Supplement: Supplementary file 1 [file ijms-24-02811-s001.zip › Supplementary material/Figure_S4.pdf]

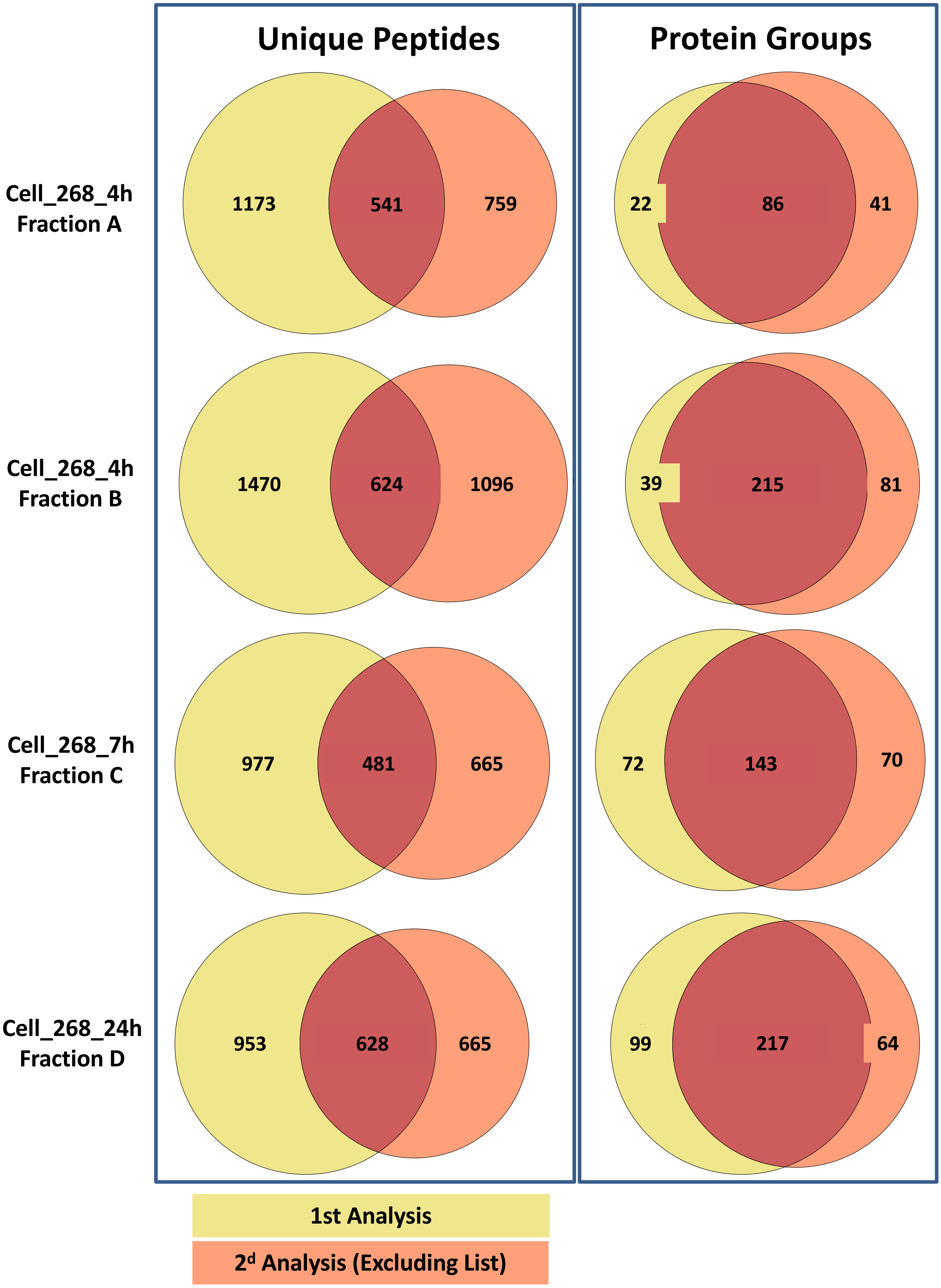
Unique Peptides
Protein Groups
Cell_268_4h
Fraction A
1173
541
759
22
86
41
Cell_268_4h
Fraction B
1470
624
1096
39
215
81
Cell_268_7h
Fraction C
977
481
665
72
143
70
Cell_268_24h
Fraction D
953
628
665
99
217
64
1st Analysis
2d Analysis (Excluding List)
